# A new cost-utility analysis assessing risk factor-guided prophylaxis with palivizumab for the prevention of severe respiratory syncytial virus infection in Italian infants born at 29–35 weeks’ gestational age

**DOI:** 10.1371/journal.pone.0289828

**Published:** 2023-08-10

**Authors:** Ian P. Keary, Roberto Ravasio, John R. Fullarton, Paolo Manzoni, Marcello Lanari, Bosco A. Paes, Xavier Carbonell-Estrany, Eugenio Baraldi, Jean-Éric Tarride, Barry Rodgers-Gray

**Affiliations:** 1 Violicom Medical Limited, Aldermaston, Berkshire, United Kingdom; 2 MA Provider, Milan, Lombardy, Italy; 3 Department of Public Health and Pediatric Sciences, University of Torino School of Medicine, Turin, Piedmont, Italy; 4 Division of Paediatrics and Neonatology, Degli Infermi Hospital, Ponderano, Italy; 5 Paediatric Emergency Unit, IRCCS-Policlinico Ospedaliero-Universitario di Bologna, Bologna, Italy; 6 Department of Pediatrics (Neonatal Division), McMaster University, Hamilton, Ontario, Canada; 7 Neonatology Service, Hospital Clinic, Barcelona, Catalonia, Spain; 8 Department of Women’s and Children’s Health, University Hospital of Padova, Veneto, Italy; 9 Institute of Pediatric Research, "Città della Speranza", Padova, Veneto, Italy; 10 Programs for Assessment of Technology in Health (PATH), The Research Institute of St. Joe’s Hamilton, St. Joseph’s Healthcare, Hamilton, Ontario, Canada; The University of Texas MD Anderson Cancer Center, UNITED STATES

## Abstract

Since the last Italian cost-utility assessment of palivizumab in 2009, new data on the burden of respiratory syncytial virus (RSV) and an International Risk Scoring Tool (IRST) have become available. The objective of this study was to provide an up-to-date cost-utility assessment of palivizumab versus no prophylaxis for the prevention of severe RSV infection in otherwise healthy Italian infants born at 29–31 weeks’ gestational age (wGA) infants and those 32–35wGA infants categorized as either moderate- or high-risk of RSV-hospitalization (RSVH) by the IRST. A decision tree was constructed in which infants received palivizumab or no prophylaxis and then could experience: i) RSVH; ii) emergency room medically-attended RSV-infection (MARI); or, iii) remain uninfected/non-medically attended. RSVH cases that required intensive care unit admission could die (0.43%). Respiratory morbidity was considered in all surviving infants up to 18 years of age. Hospitalization rates were derived from Italian data combined with efficacy from the IMpact-RSV trial. Palivizumab costs were calculated from vial prices (50mg: €490.37 100mg: €814.34) and Italian birth statistics combined with a growth algorithm. A lifetime horizon and healthcare and societal costs were included. The incremental cost-utility ratio (ICUR) was €14814 *per* quality-adjusted life year (QALY) gained in the whole population (mean: €15430; probability of ICUR being <€40000: 0.90). The equivalent ICURs were €15139 *per* QALY gained (€15915; 0.89) for 29–31wGA infants and €14719 *per* QALY gained (€15230; 0.89) for 32–35wGA infants. The model was most sensitive to rates of long-term sequelae, utility scores, palivizumab cost, and palivizumab efficacy. Palivizumab remained cost-effective in all scenario analyses, including a scenario wherein RSVH infants received palivizumab without a reduction in long-term sequelae and experienced a 6-year duration of respiratory morbidity (ICUR: €27948 *per* QALY gained). In conclusion, palivizumab remains cost-effective *versus* no prophylaxis in otherwise healthy Italian preterm infants born 29–35wGA. The IRST can help guide cost-effective use of palivizumab in 32–35wGA infants.

## Introduction

Respiratory syncytial virus (RSV) is a major cause of respiratory illness. Globally, it is estimated that 12.9 million lower respiratory tract infections (LRTI), 2.2 million hospitalizations and >66,000 deaths in children <1 year are attributable to the virus each year [1]. Premature infants are well-recognized as being at increased risk of severe RSV-LRTI and the need for hospitalization [2]. Although increasing gestational age mitigates this risk, those infants born at 32–35 weeks’ gestational age (wGA) remain at high-risk of hospitalization due to the combined effects of intrinsic (*e*.*g*. pulmonary immaturity) and extrinsic (*e*.*g*. presence of siblings) risk factors [3–7]. Approximately 4% of 32–35wGA infants are hospitalized for RSV (RSVH) [8].

Passive immunoprophylaxis with palivizumab is currently the only intervention that is widely available to prevent RSV-LRTI in high-risk infants, including premature infants born at ≤35wGA. Nirsevimab will provide an additional option for immunoprophylaxis, but whilst it has been recently approved in several countries, it is not yet available for clinical use. In Italy, a previous health economic analysis published in 2009 demonstrated palivizumab to be cost-effective *versus* no prophylaxis in premature infants without bronchopulmonary dysplasia from the Italian National Health Service perspective (ICUR *per* QALY gained of €9380 in <33wGA infants and €14937 in 33–35wGA infants) [9]. Palivizumab reimbursement in Italy is currently available for preterm infants born <29wGA and ≥29wGA, aged <12 months and >6 months, respectively, at the start of the season [10]. However, this has not always been the case; in 2016, the Italian Medicines Agency (Agenzia italiana del farmaco; AIFA) restricted reimbursement to the more premature group <29wGA, before revoking this decision the following year [10, 11].

Since the publication of the Italian cost-analysis of palivizumab [9], there have been several key developments with implications for the re-assessment of the cost-effectiveness of interventions to prevent RSV infection. Perhaps most significantly, an International risk scoring tool (IRST) has been developed and validated as a means of identifying those 32–35wGA infants at greatest risk of RSVH [12]. Moreover, recent studies have provided additional perspectives on the burden of RSV beyond the need for acute hospitalization. The temporal association between severe RSV infection in young children and the potential for long-term childhood and adolescent respiratory morbidity [13–16] and obstructive lung-function in adulthood [17, 18], has been reinforced. Furthermore, the recognition of the burden of medically attended RSV not requiring hospital admission (MARI) has increased, giving rise to additional data on this parameter [6, 19, 20]. The objective of this study was to undertake a new cost-utility analysis of palivizumab *versus* no prophylaxis in otherwise healthy Italian premature infants born at 29–35wGA, incorporating use of the IRST as well as the latest epidemiological and cost data.

## Materials and methods

This cost-utility analysis assessed the use of palivizumab *versus* no prophylaxis in all 29–31wGA infants and those 32–35wGA infants who are at moderate- and high-risk of RSV-LRTI, as determined by the IRST [12]. The analysis adopted an Italian societal perspective with both healthcare/payer and parental costs included, and is reported commensurate with the Consolidated Health Economic Evaluation Reporting Standards (CHEERS) 2022 [21].

### Systematic review

To inform the development of the current analysis, a systematic literature review was undertaken to identify the key characteristics of previous cost-effectiveness and cost-utility models (S1 Appendix). This included assessment of the modelling methodologies utilized, the model structures adopted, key parameters, the most influential variables, and the use of innovative approaches to handle important calculations, such as infant weight at the time of palivizumab dosing.

### Model structure

**Fig 1:**
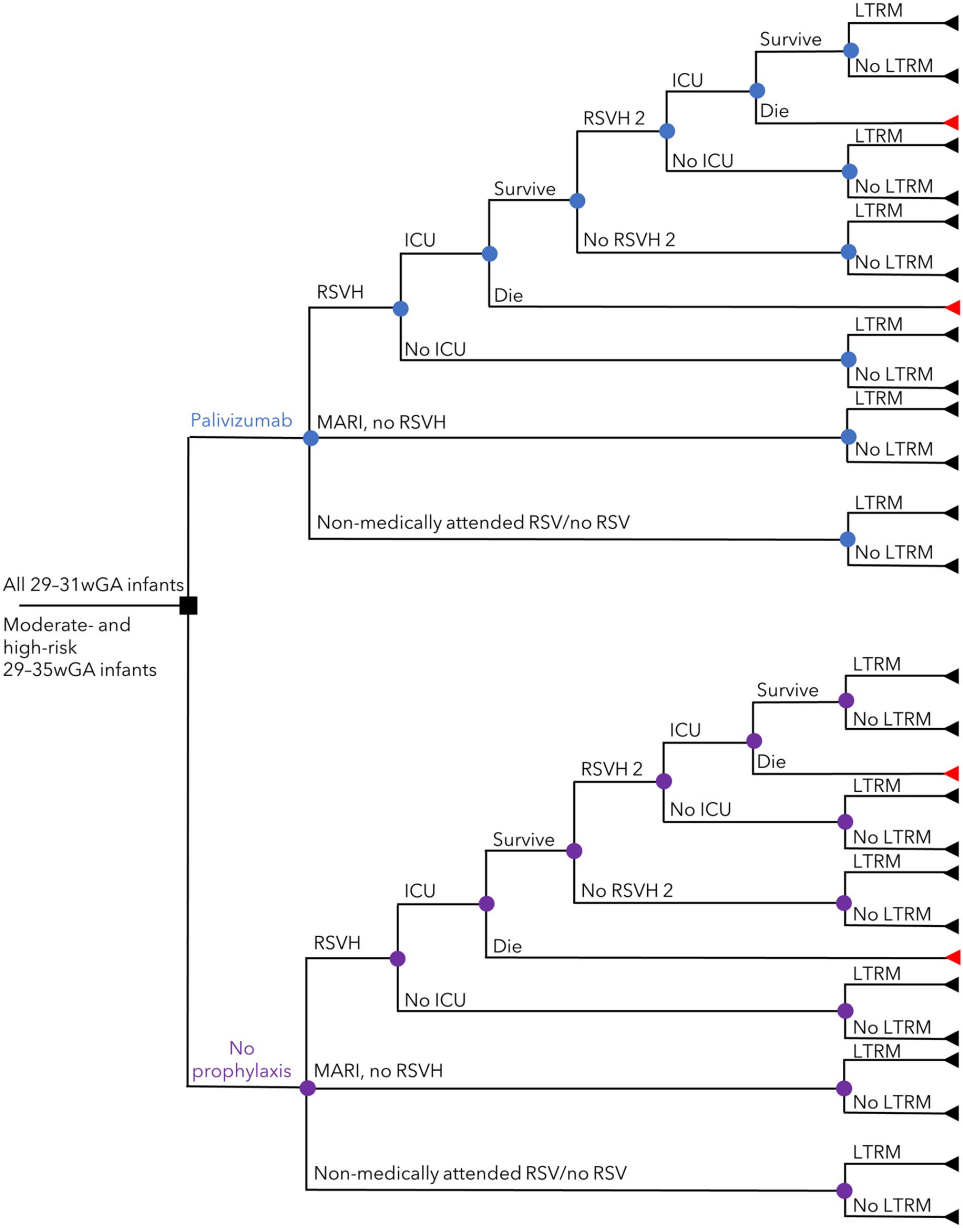
Decision tree describing the clinical pathway used in the model. Nodes represent points where more than one event is possible; the square node represents the decision addressed by the model. Branches represent the possible events that patients may experience. Triangles represent the decision tree endpoints. *ICU* intensive care unit, *LTRM* long-term respiratory morbidity, *MARI* medically-attended RSV infection without hospitalization, *RSV* respiratory syncytial virus, *RSVH* RSV-related hospitalization.

A lifetime decision-tree model was developed wherein prophylaxed/non-prophylaxed infants had RSVH, MARI, or remained uninfected/non-medically attended (Fig 1). Following RSVH, infants could be admitted to the intensive care unit (ICU) or not, and those admitted to the ICU were assumed to either survive or die. Survivors could go through a second identical cycle of RSVH. All surviving infants had the potential to experience long-term respiratory morbidity, for up to 18 years in the base case, over a life-time horizon. The model was predicated on an earlier model that we developed [22] that followed the International Society for Pharmacoeconomics and Outcomes Research (ISPOR) guidelines [23].

### Model assumptions and inputs

#### Palivizumab efficacy

The efficacy of palivizumab was drawn from the Impact-RSV study [24] the RSVH rates for non-prophylaxed *versus* prophylaxed infants being 4.9% *versus* 1.8% and 10.1% *versus* 1.8% in 29–31wGA and 32–35wGA infants, respectively. These rates translate to relative risk reductions of 63.3% and 82.2%. The effect of palivizumab on the incidence of respiratory morbidity was calculated by generating a curve of best fit using data from Simoes *et al*. 2007 [25], Blanken *et al*. 2013 [26] and Yoshihara *et al*. 2013 [27], as previously described by Sanchez Luna *et al*. 2017 [28].

#### RSV-related hospitalization (RSVH)

The RSVH rate for 29–31wGA infants not receiving palivizumab was 5.88%, and was abstracted from the study by Priante *et al*. 2019 [29] which assessed RSVH rates in Italian infants born at 29–35wGA (Table 1). For 32–35wGA infants not receiving palivizumab, data from the IRST were used, since this dataset excluded infants that received prophylaxis and provided risk-group specific RSVH rates [12]. The RSVH rates in those infants receiving prophylaxis were calculated by applying the efficacy of palivizumab (63.3%, 82.2%) [24] to the non-prophylaxed rates (Table 1). Ward length of stay (LOS) was based on experience from clinical practice in Italy (“Manzoni P. [Unpublished]” Personal Communication. 12^th^ December 2022), with the same duration (mean 6.8 days) applied to both prophylaxed and non-prophylaxed infants. ICU admission rates (palivizumab: 8.7%; no palivizumab: 20.0%) were calculated from previous studies [30, 31]. For any infants admitted to ICU, a mortality rate of 0.43% was applied [32, 33].

**Table 1:** Input parameters associated with RSVH and MARI in the base case.

Parameter	Point estimates		Reference source(s)
	Palivizumab	No Palivizumab	
**Palivizumab efficacy (RRR)**			
• 29–31wGA	63.3%	-	Notario *et al*. 2014 [24]
• 32–35wGA	82.2%	-	
**RSVH** ^a^			
• 29–31wGA	2.16%	5.88%	Priante *et al*. 2019 [29]
• 32–35wGA moderate and high-risk (base case)	1.75%	6.30%	Blanken *et al*. 2018 [12]
• Ward LOS, mean days	6.8	6.8	“Manzoni P. Unpublished.” Personal Communication. 12^th^ December 2022.
• ICU rate	8.7%	20.0%	Calculated from Ravasio *et al*. 2006 [30] and Cutrera *et al*. 2019 [31]
• Utility in hospital	0.60	0.60	Weiner *et al*. 2012, [34] Leidy *et al*. 2005 [35]
• Utility post discharge			
• No sequelae	0.88	0.88	Greenough *et al*. 2004 [36]
• Long-term sequelae	0.79	0.79	Chiou *et al*. 2005 [37]
• Mortality^b^	0.43	0.43	Wang *et al*. 2008 [32] & 2011 [33]
**MARI**			
• 29–31wGA	4.26%	11.60%	Notario *et al*. 2014, [24] Carbonell *et al*. 2010 [19]
• 32–35wGA	2.95%	16.57%	Greenough *et al*. 2004 [36]
- Utility	0.95	0.95	
**MARI/No RSV** ^c^			
• Utility			
• No sequelae or respiratory symptoms	0.95	0.95	Greenough *et al*. 2004 [36]
• Long-term sequelae or respiratory symptoms	0.79	0.79	Chiou *et al*. 2005 [37]

^a^First and subsequent RSVHs.

^b^Applied only to patients in ICU.

^c^Infants without an RSV infection or not requiring medical management.

*ICU* intensive care unit, *LOS* length of stay, *MARI* medically-attended RSV infection without hospitalization, *RRR* relative risk reduction, *RSV* respiratory syncytial virus, *RSVH* RSV-related hospitalization, *wGA* weeks’ gestational age.

#### International Risk Scoring Tool (IRST)

The IRST was developed using logistic regression analysis of a pooled dataset of 13,475 infants born 32-35wGA drawn from six observational studies [12]. Of the 13,475 infants, 484 (3.6%) had an RSVH at ≤1 year (S1 Table) [12]. The final version of the IRST included three risk factors: birth 3 months before to 2 months after the season start date; household smoking and/or smoking while pregnant; and siblings (excluding those from multiple births) and/or day care attendance (S1 Fig). A score of ≤19 was considered low-risk for RSVH (RSVH rate: 1.0%), scores of 20–45 considered moderate-risk (3.3%), and scores of 50–56 high-risk (9.5%) (S2 Table). The area under the receiver operating characteristic curve (AUROC) was 0.773 (sensitivity 68.9%; specificity 73.0%) [12].

#### Medically-attended RSV infection (MARI)

MARI rates were taken from the palivizumab arm of the motavizumab trial [19] wherein it was assumed that 4.26% of 29–31wGA and 2.95% of 32–35wGA infants receiving prophylaxis would present at hospital but not be admitted. In line with the Italian healthcare system, it was assumed that these visits would be to the emergency department. For non-prophylaxed infants, the rates of MARI were adjusted according to the rate of palivizumab efficacy used for each wGA group.

#### Long-term respiratory morbidity

A previously published algorithm by Sanchez-Luna *et al*. 2017 [28] was used to calculate the effect of palivizumab on long-term respiratory sequelae [28]. The algorithm used the Spanish SPRING study [38] in combination with three palivizumab studies [25–27] to estimate the risk reduction associated with prophylaxis *versus* no prophylaxis for 32–35wGA infants up to 6 years of age. This approach was adopted in the current model for 0–6 years of age and extended to age 18 years using data on respiratory morbidity from the Swedish prospective study by Sigurs *et al*. [13, 39–41]. To adjust for differences in baseline respiratory morbidity rates between the cohorts, the Sigurs *et al*. data [13, 39–41] were fitted to the lower rates observed in the premature-specific SPRING study [38]. This approach was chosen because the Sigurs *et al*. data [13, 39–41] included infants born both preterm and term with a RSVH, and their matched controls, thus providing a less specific estimate than the SPRING study [38].

Following RSVH or MARI, any long-term respiratory morbidity was assumed to continue until 18 years of age, although with lower rates for the latter children (S3 Table). In those infants without an RSV infection or who had a non-medically attended RSV infection, respiratory morbidity was assumed to last for 6 years, using the same rates as for MARI.

#### Utilities

To account for the impact of RSVH on infants’ quality of life (QoL), a utility value of 0.60 was applied for the duration of the RSVH (6.8 days). This was extracted from a US cost-analysis [34], which in turn derived the value from a prospective study of 46 children born ≤35wGA with RSVH and 45 chronologic and gestationally age-matched controls [35] (Table 1). After discharge, a utility value of 0.88 was applied, as *per* a UK study of premature infants (median 27wGA) with chronic lung disease and RSVH (and a control group without RSVH) until 5 years of age [36]. For non-hospitalized infants, the control group utility value (0.95) from the same study [36] was used. These utility values were applied until 5 years of life, after which perfect health was assumed unless death occurred. In the case of mortality, a utility value of 0 was assigned for subsequent years.

In the absence of any RSV-specific utility data on the impact of long-term respiratory morbidity on QoL, a value of 0.79 was adopted. This was based on a study of children with mild-and-moderate asthma symptoms [37].

#### Costs

Costs were calculated on an individual level and reflected the costs of RSVH, MARI, ICU admission, the ongoing costs of respiratory morbidity, and societal costs as well as the acquisition and administration of palivizumab in Italy (Table 2). All costs were taken from publicly available sources and were adjusted, as necessary, for inflation as of January 2023. In line with Italian recommendations, a 3.0% discount was applied to costs and utilities [42]. The cost of treatment (assuming no vial sharing, and 100% adherence) was calculated using publicly available information on the price of palivizumab (50mg: €490.37; 100mg: €814.34) [43] and the least expensive combination of vials needed based on infant weight. Using available data from the Italian Society of Neonatology, average birthweight was estimated to be 1383g and 2203g for 29–31wGA and 32–35wGA infants, respectively [44]. Assuming an even distribution of births across the year, infant weight at palivizumab administration was modelled using the growth algorithm described by Narayan *et al*. 2020 [45]. Based on this approach, the average cost of palivizumab in the base case was estimated to be €2,978 *per* 29–31wGA infant and €3,422 *per* 32–35wGA infant. Administration by a nurse was estimated to cost an additional €86.98 *per* infant based on the cost of a general outpatient appointment [46] and an average of 4.21 injections *per* infant (“Manzoni P. [Unpublished]” Personal Communication. 12^th^ December 2022).

**Table 2:** Direct and indirect costs in the base case.

Parameter	Cost (€EUR)	Units	Reference source(s)
**Healthcare/payer (direct) costs**			
**Palivizumab**			
• 50 mg vial	490.37	Lowest combination of vials *per* infant weight	[Veneto Region PVZ Guidelines] [43]
• 100 mg vial	814.34		
• Administration	20.66	1 *per* injection	Italian National Tariff [46]
**Preadmission healthcare contact**	241.05	1 *per* RSVH^b^	Italian Ministry of Health [47]
**RSVH total stay (excluding ICU)**	5768.00	1 *per* RSVH^b^	Italian National Tariff [46]
**ICU total stay**	11179.00	1 *per* ICU admission	Italian National Tariff [46] (Mean cost of ventilation <96h and >96h)
**MARI**			
• ED visit	241.05	1 *per* affected infant^b^	Italian Ministry of Health [47]
**Respiratory morbidity (*per* annum)**	1254.72	1 *per* affected infant^c^	Dal Negro *et al*. 2016 [48]
**Societal (indirect) costs**			
**Palivizumab administration**			
• Transport	64.58	1 *per* infant receiving palivizumab	Automobile Club of Italy (€/km) [49], based on representative small family car, assumes 30 km round trip *per* palivizumab injection
• Missed work	85.25		Italian National Institute of Statistics [50], assumes 3 hours missed *per* injection for 50% injections
**RSVH**			
• Missed work	577.80	1 *per* infant with RSVH^b^	Mitchell *et al*. 2017 [51] & Italian National Institute of Statistics [50]
• Childcare^a^	58.30		Mitchell *et al*. 2017 [51]
• Transport^a^	62.73		Mitchell *et al*. 2017 [51]
• Other out of pocket expenses^a^	171.72		Mitchell *et al*. 2017 [51]
**MARI attendance**			
• Transport	15.34	1 *per* infant with MARI^b^	Automobile Club of Italy (€/km) [49], based on representative small family care, assumes 30 km round trip *per* palivizumab injection
• Missed work	20.25		Italian National Institute of Statistics [50]
**Loss of earnings following death**	617313.62	1 *per* infant suffering mortality^b^	Italian National Institute of Statistics [50, 52], statista.com [53]

^a^Costs adjusted to Italian 2023 levels using Italian Consumer Price Index [54] and Purchasing Power Parities [55], January 2022.

^b^See Table 1 for rates.

^c^See S3 Table for rates.

*ED* emergency department, *ICU* intensive care unit, *MARI* medically-attended RSV infection without RSVH, *RSV* respiratory syncytial virus, *RSVH* RSV-related hospitalization.

#### Adjustment for number of births in each age group

To account for fewer births in the 29–31wGA group than in the 32–35wGA, calculations were weighted on the basis that 25.06% of the total cohort of interest would be 29–31wGA infants and 74.94% would be 32–35wGA infants. This was based on Canadian birth statistics over the years 2016–2020, in the absence of publicly available Italian data [56]. The proportion of high- and moderate-risk 32–35wGA infants was assumed to be 48.7% in line with Blanken *et al*. 2018 [12].

### Model outputs

As recommended by established guidelines [57–59], outcomes were expressed as the incremental cost *per* QALY gained (ICUR) when palivizumab was compared to no intervention. A QALY is a summary outcome measure that combines the impact of an intervention in terms of both the quality and quantity of life; a value of 1 indicates a year of perfect health; lower scores represent reduced life expectancy and/or reduced quality of life.

### Sensitivity analyses

Commensurate with best practice, [60] uncertainty was assessed using deterministic (DSA) and probabilistic (PSA) sensitivity analyses. As measures of dispersion were absent in the majority of source materials, analyses employed limits of plus or minus 10% (PSA) and 20% (DSA) on the values of the tested variables (wider limits were used in the DSA to help identify variables with non-linear effects). The DSA results were used qualitatively to rank the variables and identify those with the most influence. In the PSA (10,000 Monte Carlo simulations), gamma distribution was used as the default for costs, beta distribution for utilities and hospitalization and associated rates, and normal distribution for discount rates and mortality (as an approximation of Poisson distribution) rate (S4 Table). Although often regarded as static, given the high levels of economic uncertainty at the time of writing and to account for changing rates over the accounting period, we chose to include discounting in the PSA. To supplement these analyses, a probabilistic DSA was also undertaken based upon the methodology of Vreman *et al*. [61] The default number of probabilistic repetitions *per* plotted point was 1000.

Cost-effectiveness acceptability curves (CEACs) were used to represent the uncertainty. The CEAC presents the probability of palivizumab to be cost-effective against no prophylaxis at different willingness to pay thresholds *per* QLAY gained.

### Scenario analyses

Various scenario analyses were undertaken. These included: i) the exclusion of indirect/societal costs ii) removal of discounting; iii) inclusion of vial sharing with 5% wastage (NB palivizumab vials are single use only); iv) reduction of the duration of respiratory morbidity to 13 or 6 years; v) standardization of hospitalization rate in non-prophylaxed infants based on data from Priante *et al*. 2019 [29]; vi) estimation of age-group specific birth weight; vii) use of palivizumab list price in place of published price; viii) no benefit of palivizumab in reducing long-term respiratory morbidity in infants with RSVH combined with 6-year duration of long-term respiratory morbidity in affected non-hospitalized infants.

### Model validation

The model structure, data and assumptions were validated *via* consultation with acknowledged clinical experts in RSV (XCE, BP, PM, EB and ML) and comparison with previous cost-analyses of palivizumab identified during the systematic review (S1 Appendix). Model coding was validated by comparison to an alternative model that was constructed independently from the main model but based on an identical structure, assumptions, and data. Finally, for 32–35wGA infants, the non-prophylaxed arm of the model was compared to the Spanish FLIP-2 [4] (Risk Factors Linked to RSV Infection Requiring Hospitalization in Premature Infants) population of 5,441 infants born at 32–35wGA, and the resulting outcomes (predicted *versus* actual) compared: number of RSVHs; ICU admittance; and mortality rate. As FLIP-2 considered all 32–35wGA infants, the total population, regardless of risk status, was used for validation purposes.

### Software

The model was developed using Microsoft Excel 365.

## Results

Palivizumab prophylaxis of all 29–31wGA infants and those 32–35wGA infants identified as moderate- or high-risk for RSVH by the IRST, was associated with increased treatment and indirect costs but led to a reduction in direct healthcare costs and improved QoL compared to no prophylaxis (Table 3). In the base case, the ICUR was €14814 *per* QALY gained when palivizumab was compared with no prophylaxis. When considering 29–31wGA and 32–35wGA infants separately, the corresponding ICURs were €15139 and €14719 *per* QALY gained, respectively.

**Table 3:** Incremental costs and utilities for palivizumab *versus* no prophylaxis using the IRST to identify moderate- and high-risk 32–35wGA infants.

RST	29–35wGA		29–31wGA		32–35wGA	
	No PVZ	PVZ	No PVZ	PVZ	No PVZ	PVZ
Treatment costs (€)	0	3348	0	3020	0	3458
Direct costs (€)	1751	419	1657	482	1782	399
Indirect costs (€)	37	155	40	161	36	153
Difference in costs (€)						
Including indirect costs	2135		1966		2192	
*Excluding indirect costs*	*2017*		*1845*		*2075*	
QALYs	30.814	30.958	30.825	30.955	30.811	30.959
Difference in QALYs	0.144		0.130		0.149	
**ICUR (€) *per* QALY gained**						
**Including indirect costs (base case)**	**14814**		**15139**		**14719**	
***Excluding indirect costs***	***13995***		***14210***		***13933***	

*ICUR* incremental cost utility ratio, *IRST* international risk scoring tool, *QALY* quality adjusted life year, *RST* risk scoring tool, *wGA* weeks’ gestational age.

### Sensitivity analyses

**Fig 2:**
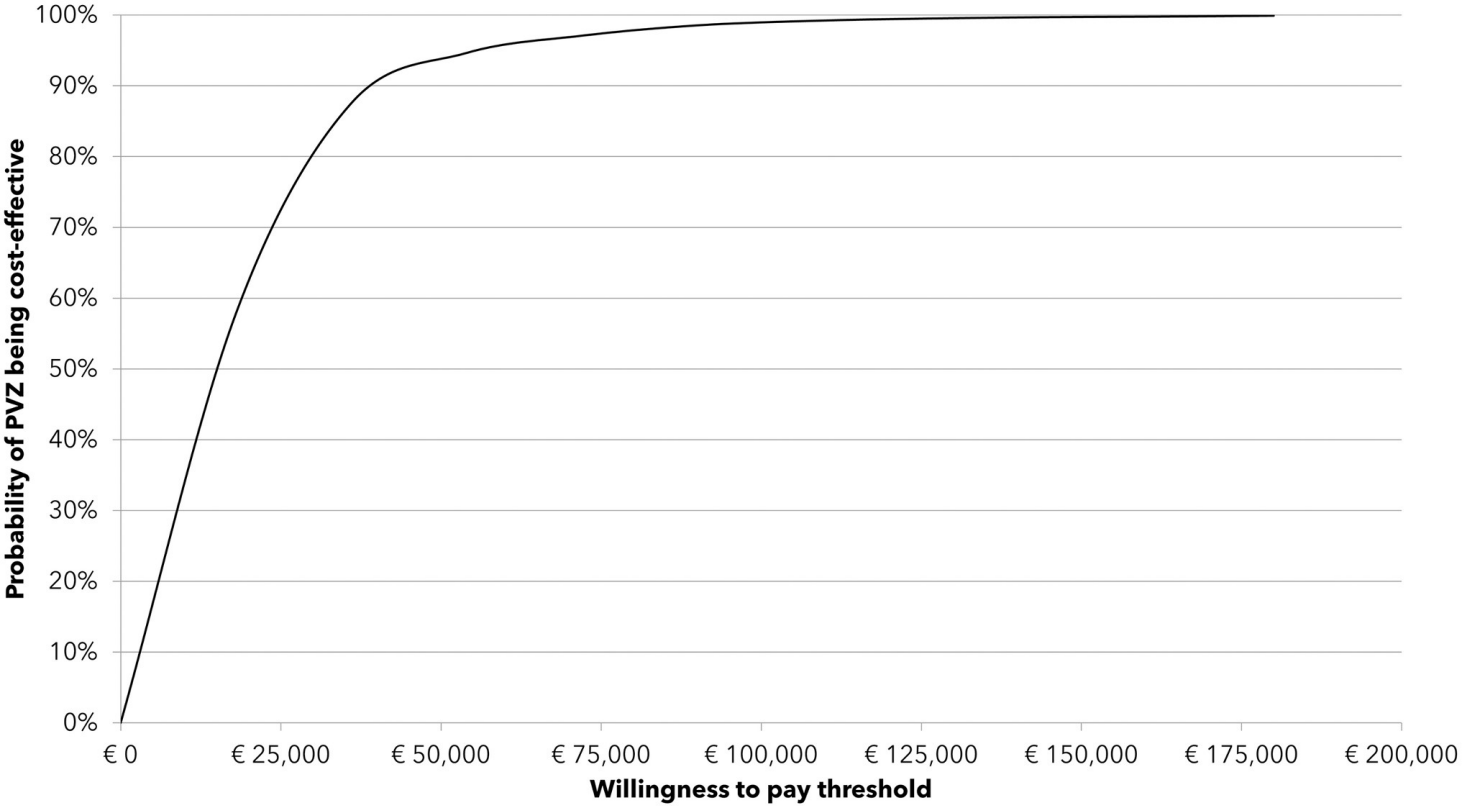
Cost-effectiveness acceptability curve for palivizumab prophylaxis *vs* no prophylaxis) in Italian preterm infants born at 29–35wGA. Results are based on the probabilistic analysis after 10,000 Monte Carlo simulations.

A mean ICUR of €15430 *per* QALY gained was predicted with palivizumab *versus* no-prophylaxis for the 29–35wGA group, with 89.9% of PSA runs falling below a €40000 *per* QALY willingness to pay (WTP) threshold (Fig 2). The mean ICURs *per* QALY gained in the individual age groups were €15915 and €15230 in the 29–31wGA and 32–35wGA group, respectively. The DSA found the model to be most sensitive to long-term morbidity rates, utility scores, palivizumab cost (100mg), palivizumab efficacy and average birth weight of 32–35wGA infants (S2 Fig).

The probabilistic DSA results indicated that the 10 most individually influential variables identified by the DSA responded broadly linearly, except for palivizumab efficacy in the 32–35wGA infants, which produced an increasingly positive impact on the ICUR when increased above the default value (S3 Fig). The probabilistic DSA results suggest that the qualitative DSA observations on the relative impact of these variables are valid.

### Scenario analyses

Exclusion of societal costs slightly improved the ICUR (€13995 *versus* €14814 *per* QALY gained in 29–35wGA infants, Table 3). Removal of discounting had a larger impact and improved the ICUR to €11280 *per* QALY gained in the full population (Table 4). Likewise, consideration of vial sharing with 5% wastage resulted in an ICUR of €9532 *per* QALY gained, a considerable improvement *versus* the base case. Conversely, reducing the period of long-term respiratory morbidity to 13 and 6 years increased the ICUR to €19193 and €27381 *per* QALY gained, respectively. Combining removal of the beneficial impact of palivizumab on the rate of long-term respiratory morbidity in infants with RSVH and a 6-year duration of respiratory morbidity, increased the ICUR to €27948 *per* QALY gained. The remaining scenarios all had a relatively minor impact.

**Table 4:** Results of scenario analyses.

	ICUR *per* QALY gained (€)	
Scenario	As *per* scenario	Base case
No discounting of costs or utilities	11280	14814
*Base case*: *1*.*5% discount*		
Vial sharing (5% wastage)	9532	
*Base case*: *No vial sharing*		
13-year respiratory morbidity	19193	
*Base case*: *18 years*		
6-year respiratory morbidity	27381	
*Base case*: *18 years*		
Italy-specific RSVH rate (5.88%) in 32–35wGA	15274	
*Base case 6*.*3%*		
Adjusted age group-specific birthweight 29–31wGA: 1441.12g, 32–35wGA: 1907.51g	14205	
*Base case*: *29–31wGA*: *1382*.*98g*, *32–35wGA*: *2203*.*43g*		
Use of palivizumab list price (50mg vial: €544.86; 100mg vial €904.82) in place of the published (discounted) price	17329	
*Base case*: *50mg vial*: *€490*.*37; 100mg vial €814*.*34*		
No benefit of palivizumab in reducing long-term respiratory morbidity in RSVH infants combined with 6 years duration of long-term respiratory morbidity in affected infants	27948	
*Base case*: *18-year respiratory morbidity and a reduction in rate of long-term respiratory morbidity with palivizumab in RSVH and non RSVH infants as per* S3 Table		

*ICUR* incremental cost-utility ratio, *RSVH* respiratory syncytial viral hospitalization, *wGA* weeks’ gestational age

### Model validation

No marked differences between the model predictions and actual outcomes of FLIP-2 [4] were found for number of RSVHs (201 *versus* 202, respectively), ICU admittance (20% *versus* 17.9%), and mortality rate (0.003% *versus* 0%).

## Discussion

Preterm infants born at 29–35wGA are an established group at high-risk of severe RSV illness [4–8]. Despite this, recommendations on the use of palivizumab to prevent RSVH vary widely in national and regional guidelines and associated reimbursement policies [62–64]. In Italy, palivizumab reimbursement for infants born at >29wGA was withdrawn in 2017, only to be reinstated the following year due to an increase in RSVH [10, 11]. Given this procedural variation in reimbursement, an up-to-date, country-specific analysis of the cost-utility of palivizumab is of critical importance to evaluate its ongoing role in clinical practice. This cost-analysis evaluation in Italian preterm infants, is the first to incorporate variables inclusive of the IRST, the consideration of MAR,I and the potential for respiratory morbidity extending throughout childhood. Together with Italian costs and model inputs, prophylaxis of all infants born at 29–31wGA and those 32–35wGA infants identified as moderate or high-risk of RSVH by the IRST was found to be cost-effective, with an ICUR of €14814 *per* QALY gained (mean ICUR: €15430 *per* QALY). This is approximately €25000 below the €40000 *per* QALY WTP threshold in Italy, as identified by the Italian Health Economics Association [65]. Prophylaxis was slightly more cost-effective in the moderate- and high-risk 32–35wGA infants than in 29–31wGA infants (ICURs: €14719 *versus* €15139 *per* QALY gained, respectively), illustrating the benefits in the former group of targeting palivizumab use *via* the IRST. Prophylaxis with palivizumab remained cost-effective *versus* no intervention in all scenarios examined, and for the high and moderate risk groups when assessed individually (S5 Table).

In a previous Italian analysis that considered comparable age groups and model structures [9], the costs *per* QALY gained *per* sub-population of interest were €14937 in 33-35wGA infants and €9380 in <33wGA infants. Analogous to our model, it considered initial and subsequent hospitalizations, ICU admission, mortality and respiratory morbidity. However, despite the similarities in overall results, any comparison must also account for changes in costs over time, differences in model parameters and rates along with structural differences, such as the inclusion of indirect costs and MARI. As such, comparison of the two models provides an ideal framework to assess the key characteristics of our updated model.

In the previous Italian analysis [9] the authors included higher RSVH rates in non-prophylaxed infants than in our model (10.3% and 9.8% *versus* 5.9% and 6.3% for the <33/29–31wGA and 33–35/32–35wGA groups, respectively) and assumed a higher rate of palivizumab efficacy in reducing RSVH, particularly in the more premature infants (80.5% and 84.5% *versus* 63.3% and 82.2%). Their reported ICU admission rates were 3% and 1% in non-prophylaxed and prophylaxed infants, respectively, *versus* 20.0% and 8.7% of those hospitalized in our model [9]. The RSVH and palivizumab efficacy rates used in the previous Italian analysis [9] were drawn from a 2002 meta-analysis [66], while the ICU rates were from the IMpact-RSV study conducted in 1998 [67], neither of which included infants from Italy and were dominated by US infants. In contrast, the more conservative RSVH rates in non-prophylaxed infants used in our study were drawn from Priante *et al*. 2019 [29], which provided a real-world, Italy-specific estimate of RSVH rates in infants born at 29–35wGA and Blanken *et al*. 2018 [12] which detailed the IRST and the RSVH rate associated with each risk category based on data from >13,000 infants from various Northern Hemisphere countries, including Italy. The palivizumab efficacy rate utilized in our model was taken from a *post hoc* analysis of the IMpact-RSV study (published in 2014) that provided age group-specific data and was not available to the authors of the previous analysis [24]. Furthermore, rates of ICU admission were calculated from Italian sources [30, 31], including the 2019 study by Cutrera *et al*. [31] of RSV admissions to 6 Italian paediatric institutes and 15 Italian Paediatric Intensive Care units over the course of 3 RSV seasons. The mean length of stay for an RSVH was drawn from real-world, clinical experience in Sant’Anna Hospital in Turin, Italy. We believe use of these data derived directly from clinical practice when available or from age-group-specific analysis of clinical trial data are the most representative of the current situation in Italy.

Mortality rates in our study were also markedly more conservative than used in the previous Italian analysis, reflecting the increasing recognition that death due to RSV is a rare event in otherwise healthy preterm infants in the modern healthcare setting. Our study assumed a mortality rate of 0.43% and applied it only to those infants admitted to the ICU, whereas the previous Italian analysis [9] applied 4% mortality to hospitalized infants and 0.8% to non-hospitalized infants. When calculated across the whole population, our model predicted a smaller absolute difference in mortality rate in the whole population of 0.004% in the 29–31wGA group and 0.005% in the 32–35wGA group *versus* 0.27% in the previous Italian model. Of note, our study utilized available Italian data to determine the birthweight (29–31wGA: 1392g; 32–35: 2203g) and average number of treatment visits *per* infant (4.21), while palivizumab cost (29–31wGA: €2977.97, 32–35wGA: €3422.15) was determined based on the amount of palivizumab *per* injection calculated according to predicted growth. This contrasts to the previous analysis which assumed a standardized palivizumab cost of €3099.84 and 5 injections for all infants [9]. The importance of birthweight in determining cost of treatment and, therefore, cost *per* QALY gained is underscored by its identification as one of the five most important variables in the DSA.

Indirect costs were found to be another potentially important consideration in the cost-utility of palivizumab. Our systematic review revealed that many previous analyses, including the one from Italy, adopted the healthcare provider perspective, and therefore excluded societal costs (S1 Appendix). In those analyses that did include indirect costs, the analysis was often limited to loss of future productivity and/or loss of parent time associated with RSVH, with only 3 studies including indirect costs associated with administration. However, the requirement for up to 5 injections places a burden on the parents of all treated patients in the shape of transport costs and time off work. In contrast, RSVH affects fewer families, but the potential parental cost burden associated with a stay in hospital for those affected is much higher. We chose to include indirect costs in our base case and examine their exclusion in a scenario analysis. Results indicated that the inclusion of indirect costs in the base case worsened the ICUR compared to their exclusion (Table 3). This is a reflection of the inclusion of comprehensively documented real-world parental costs [51] combined with the realistic RSVH rates (Table 1), and illustrates the importance of considering all indirect costs not just those associated with RSV-LRTI. More detailed information on indirect costs associated with long-term respiratory morbidity following severe RSV-LRTI is a currently unmet research need.

Another clinically relevant component that differentiates the current analysis from previous assessments of palivizumab cost-effectiveness was the inclusion of MARI. Although increasingly characterized as a significant contributor to the overall burden of RSV [6, 19, 20], none of the cost-analyses identified by the systematic review considered MARI in their model structure (S1 Appendix). This may be explained by the relatively limited data available regarding this entity to date, particularly as it relates to palivizumab use. Due to this constraint, our approach considered presentation to emergency department care only (without hospital admission), and therefore did not capture the burden of RSV experienced by primary care practitioners solely within the community. Overall, this resulted in the contribution of MARI to the overall cost being relatively small. However, MARI rates featured prominently among the ten most influential variables identified by the DSA (S2 Fig). As more data emerge, we postulate that the accuracy of our estimates of both cost and frequency will improve to better represent this important aspect of RSV illness.

Consideration of the impact of RSV beyond the initial RSV infection and/or hospitalization was addressed differently in our model and the previous Italian one. In the previous model [9], in common with most of the models identified in the systematic review (S1 Appendix), long-term respiratory morbidity was considered over a relatively short time period. Specifically, the costs of subsequent respiratory hospitalizations in the 12 months following RSVH and the costs of asthma up to 24 months were investigated; while potential differences in utilities were assessed relative to RSVH status up to 5 years of age [9]. Based on the increasingly recognized temporal association between RSV LRTI and long-term morbidity [13–16], and the available data on the beneficial effect of palivizumab on the same [25–27, 68], we adopted an approach that accounted for 18 years of respiratory morbidity and considered both the costs and utilities associated with this sequelae. Given that the DSA identified that the model was particularly sensitive to variations in long-term morbidity rates, it is reassuring to note that cost-effectiveness was maintained when the duration of the morbidity was reduced to 13 (ICUR: €19193 *per* QALY gained) and 6 years (ICUR: €27381 *per* QALY gained). Moreover, a scenario that limited the duration of the respiratory morbidity to 6 years and negated the effect of palivizumab in preventing long-term wheeze in infants with RSVH (removing any possibility of double counting the benefit of palivizumab) demonstrated that the ICUR was still well within the WTP threshold of €40000 *per* QALY at €27948 *per* QALY gained.

A key limitation of our model was the availability of data describing the short- and long-term QoL outcomes for infants born at 29–35wGA, highlighted by the fact that the DSA identified utility scores as a principal driver of the ICUR. The data utilized in the model were from Greenough *et al*. 2004 [36], which are almost two decades old but arguably remain the best available source for utilities following RSVH and are used as the basis of many published cost-effectiveness and cost-utility assessments [28, 45, 69]. Similarly, the estimate of the disutility during RSVH is over 10 years old [34]. More recent estimates of the QoL impact of RSV infection are available; however, they are less applicable to our population than the older data. For example, recent studies by Hodgson *et al*. [70] and Diez-Gandía *et al*. [71] included term and preterm children up the age of 5 years and 2 years, respectively, rather than being restricted to preterm infants, and, importantly, provided a utility for all RSV-positive children rather than a disutility specifically pertinent to children with RSVH. Utility data related to long-term morbidity following RSV-LRTI are also unavailable, so a substitute value was used from a follow-up of Italian children with asthma [48], which was the same approach taken in the previous cost analysis in Italy [9] and, more recently, Spain [28]. Another limitation is that, although premature-specific data are available for long-term respiratory morbidity (SPRING [38]) and the efficacy of palivizumab [25–27] in its prevention for up to 6 years, beyond this time point more general sources (Sigurs *et al*. [13, 40, 41]) and data extrapolation were employed. As previously mentioned, specific scenario analyses provide further confidence in our model in this regard. Although gestational age-specific Italian data for the efficacy of palivizumab in the reduction of RSVH and hospital resource use were unavailable, the IMpact sub-analysis data [24] are an acceptable surrogate, abstracted from a well-conducted randomized controlled trial. The fact that the base case was well below €40000 *per* QALY (point estimate: €14814 *per* QALY gained; mean: €15430 *per* QALY gained), that the model was validated against outcomes from FLIP-2 [4], and that the results of the extensive sensitivity analyses conducted to deal with the uncertainty associated with model parameters did not change the overall findings, all afford confidence in the reliability of our results. An additional potential benefit of prophylaxis not captured in this cost-analysis includes the impact on mental health experienced by the families/caregivers of children who suffer severe RSV-LRTI [35, 51, 72–74].

## Conclusions

This analysis established that palivizumab prophylaxis of otherwise healthy preterm Italian infants born at 29–35wGA is a cost-effective strategy compared to no prophylaxis. Adoption of the IRST in 32–35wGA infants can help optimize cost-utility and reduce overall costs whilst still protecting most of these infants. Given the mounting cost pressure on healthcare resources, these results re-affirm the role of palivizumab in preventing severe RSV-LRTI and its consequences in otherwise healthy preterm infants and present a practical rationale for its ongoing use.

## Supporting information

S1 Appendix Summary report of systematic literature review. (PDF)

S1 Table Source data and predictive accuracy of the International Risk Scoring Tool. (PDF)

S2 Table Risk groups in the International Risk Scoring Tool. (PDF)

S3 Table Rates of long-term respiratory morbidity. (PDF)

S4 Table Probability distributions used in probabilistic sensitivity analysis. (PDF)

S5 Table Cost per QALY gained in moderate- and high-risk infants. (PDF)

S1 Fig International Risk Scoring Tool—Risk factors and scoring. (PDF)

S2 Fig One-way deterministic sensitivity analysis. (PDF)

S3 Fig Probabilistic DSA spider chart. (PDF)
