# Supplementary material for: A new cost-utility analysis assessing risk factor-guided prophylaxis with palivizumab for the prevention of severe respiratory syncytial virus infection in Italian infants born at 29–35 weeks’ gestational age
Source: PLoS One. 2023 Aug 10;18(8):e0289828. doi: 10.1371/journal.pone.0289828 (PMC10414677; doi:10.1371/journal.pone.0289828)
Supplement: S1 Appendix — (PDF) [file pone.0289828.s001.pdf]

A new cost-utility analysis assessing risk factor-guided prophylaxis with palivizumab for the prevention of respiratory syncytial virus infection in Italian infants born at 29-35 weeks' gestational age

PLOS ONE

Ian P. Keary, Roberto Ravasio, John R. Fullarton, Paolo Manzoni, Marcelo Lanari, Bosco A. Paes, Eugenio Baraldi, Xavier Carbonell-Estrany, Jean-Éric Tarride, Barry S. Rodgers-Gray,

Corresponding author:
Barry S Rodgers-Gray, Violicom Medical Limited, 1 Andromeda House Calleva Park, Aldermaston, United Kingdom. RG7 8AP.
barry@violicom.co.uk

Supplementary materials 1

The Cost-Effectiveness of Respiratory Syncytial Virus Prophylaxis in 29-31 Weeks' Gestational Age Preterms: Summary Report of Systematic Literature Review

Review Question

How has the health economics of palivizumab in 29^0-31^6 wGA preterms been modelled and assessed?

Search methodology

Databases

The following electronic databases were searched:

- MEDLINE (via PubMed)
- EMBASE (via Ovid)
- The Cochrane Library
- CEA Registry
- Paediatric Economic Database Evaluation (PEDE) [to December 31, 2019]

In addition, other relevant studies and evidence were identified *via* bibliographies/reference lists of key articles, review of key HTA websites (NICE, SMC, CADTH *etc*) and targeted web searches for non-indexed articles, theses and dissertations, research and committee reports, government reports *etc.* (the 'grey literature').[1] To aid in identifying grey literature, the Grey Matters tool from CADTH and Opengrey will be utilised.

Restrictions

No publication period were set on the database searches.

No language limits were set on database searches, with the caveat that English translations of at least the abstract were available.

Search Dates

The time period in which the searches were executed was January 2022.

Search Terms

The terms in Table 1 were searched in 'all fields' and combined where stated with 'Medical Subject Headings' (MeSH) in PubMed and 'Emtree Subject Headings' (ESH) in Embase:

Table 1: Search terms used in systematic review

Concept	Search Number	Search Terms	Example PubMed Results*
**Disease**	1.	RSV OR respiratory syncytial virus OR human respiratory syncytial virus [MeSH/ESH] OR bronchiolitis OR lower respiratory tract infection	69,685
**Population**	2.	((preterm OR prematur*) AND early) OR ((28 OR 29 OR 30 OR 31 OR 32) AND (gestational age OR weeks' gestational age OR wGA))	74,581
**Drug**	3.	palivizumab OR Synagis OR prophylaxis OR immunoprophylaxis OR monoclonal antibody	2,077,589
**Health Economics**	4.	cost-effectiveness OR cost analysis OR cost-utility OR cost-benefit OR economic* OR cost* OR pharmacoeconomic* OR budget* OR cost-consequence	1,458,694
	1-4 (AND)		103

The search strings were developed in line with the good practice recommendations of the International Society for Pharmacoeconomics and Outcomes Research (ISPOR) on systematic reviews with costs and cost-effectiveness[2] and assessed against the Peer Review of Electronic Search Strategies (PRESS)[3] checklist.

Inclusion and Exclusion Criteria

The condition/domain being reviewed is RSV prophylaxis in moderate-to-late preterms. The PICOS (Population, Intervention, Comparison, Outcomes and Study Design) tool was used to identify relevant references to the systematic review outcome using the inclusion and exclusion criteria in Table 2.

Table 2: PICOS framework used in systematic review

Parameter	Inclusion criteria	Exclusion criteria
**Population**	- Preterms defined as 29^0-31^6 wGA who are otherwise healthy without underlying comorbidities	- Newborns born before 29^0 wGA or after 31^6 wGA<br>- Newborns with any other pathologic health condition
**Intervention/ exposure**	- Palivizumab<br>- RSV infection	- Treatment (not prophylaxis) with palivizumab for RSV infection<br>- Non-RSV based infections
**Comparator**	- No prophylaxis<br>- RSV prevention	- None
**Outcomes**<br>- **Main**<br>- **Additional**	- Model input parameters<br>- Model structure and methods, population, ICERs/results<br>- Data sources	- Any clinical efficacy and safety outcomes
**Study Design**	- Any type of health economic study published in full or abstract form<br>- Systematic reviews and meta-analyses of health economic studies	- Non-systematic review articles, letters, pre-clinical studies, case reports, expert opinions, editorials, letters, narratives, clinical trials and observational studies
**Language**	- Health economic studies in any language	- Studies without at least an English language abstract
**Publications date**	- All dates prior to literature search	- None

Data extraction

After removal of duplicates, studies were selected for inclusion in the review using a two-phase approach:

Phase 1 - the title and abstracts of potentially relevant citations identified from the electronic searches were assessed separately by two experienced reviewers to confirm relevance and inclusion in the study according to the inclusion criteria

Phase 2 - the full texts of those citations identified as relevant in phase 1 were assessed separately by two experienced reviewers to confirm relevance and inclusion in the review according to the inclusion criteria. If a consensus could not be reached on a citation, a third senior researcher made the decision.

Data was extracted from the full-text of all relevant articles identified in Phase 2 by one reviewer, and quality checked by a second reviewer. All information was inserted into an agreed Excel data extraction table template which included the following fields: Country, Population (and Perspective), Time Horizon, Key Model Assumptions, Discount Rates, Input Parameters (including direct and indirect costs, health resource utilisation, follow-up), Outcome Measures, Cost-Effectiveness Threshold, Methodology/Model Structure, Results (e.g. ICER and cost per quality-adjusted life-year) and Funding. For cost-utility models utility parameters (including quality of life tool and value set used) will also be extracted. Study investigators were not contacted for any missing/unreported data.

Quality assessment

Studies were evaluated following the good practice recommendations of ISPOR on systematic reviews with costs and cost-effectiveness.[2] Each of the studies was assigned a quality score using the Quality of Health Economic Studies (QHES) Instrument[4] and the NICE Quality Appraisal Checklist.[5]

Overview of results

Three hundred and three unique publications were identified from the systematic literature search, of which 256 were excluded based on title and abstract, and a further 34 on review of the full publication to give 13 included studies (Figure 1). Full details of the search strategy and final results of data extraction can be found in the accompanying protocol and spreadsheet.

Of the included studies, four were systematic reviews[6-9] and nine gave details of cost-effectiveness models.[10-18] Review of the bibliographies of the systematic reviews did not identify any additional publications for inclusion.

Figure 1: PRISMA diagram

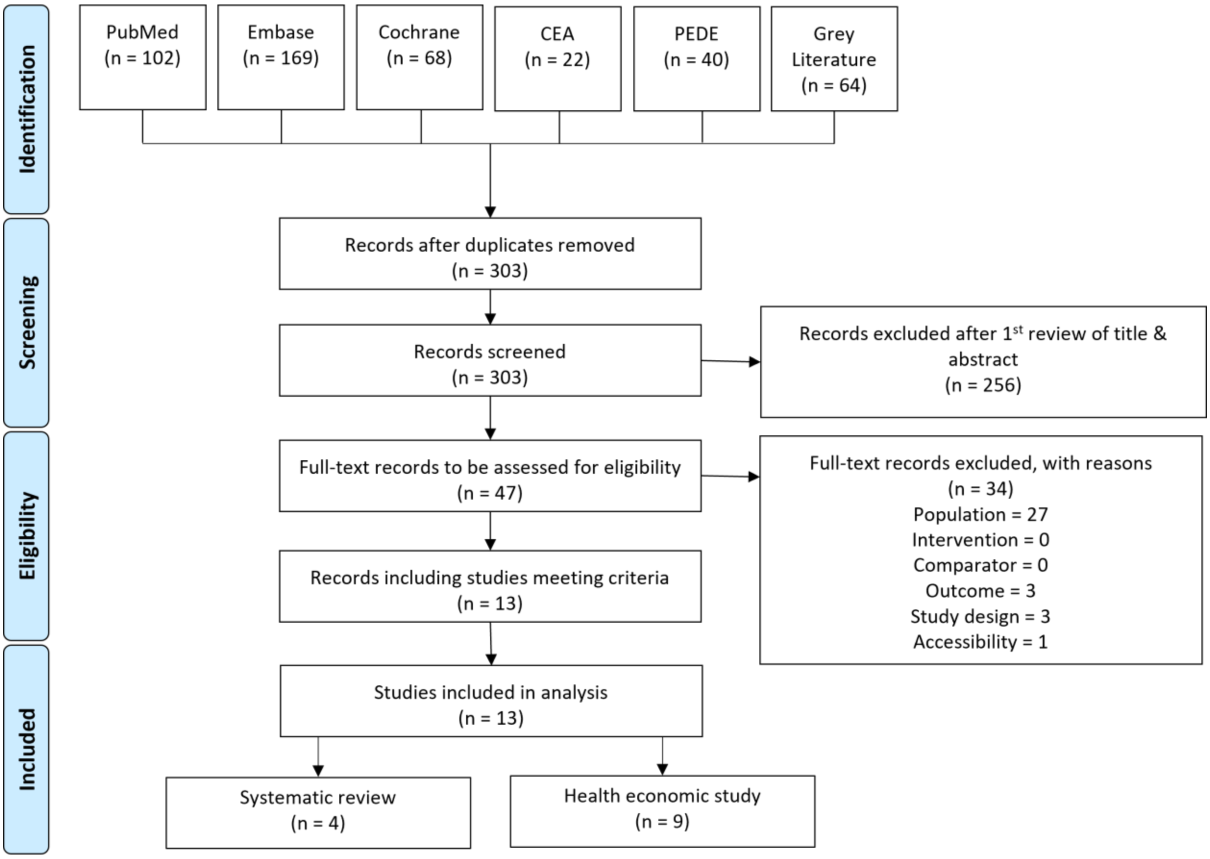

CEA: Cost-Effectiveness Analysis Registry; PEDE: Paediatric Economic Database Evaluation

Characteristics of the included cost-effectiveness studies

Summary

Of the nine publications including information on cost-effectiveness models, eight were full publications,[10-14,16-18] and one was a congress abstract.[15] As such, data abstraction from the congress abstract was limited and there was insufficient information available to perform a quality assessment.

An overview of the nine cost-effectiveness studies is as follows:

- Four studies were conducted in the UK, three in the USA and single studies in both Mexico and New Zealand
- Studies were published between 2000 and 2020
- 67% (6/9) used a decision analytic model, 22% (2/9) used a cohort study (historical and real-life) and 11% (1/9) used a Markov model

- 56% (5/9) reported on a cost-utility analysis (CUA), 22% (2/9) cost-effectiveness analysis (CEA), and the remaining 22% (2/9) combined CUA with CEA or cost-benefit analysis (CBA)
- 67% (6/9) took a payer perspective, 22% (2/9) societal perspective and 11% (1/9) both
- Time horizon ranged from 1-year to lifetime
- 33% (3/9) reported that palivizumab was cost-effective
- 56% (5/9) were funded by industry

It should be noted that of these nine studies, only two strictly included a population 29-31 wGA (or a subset thereof).[10,11] The remaining seven studies involved preterm infants up to 32 wGA, but were included in this report as this fitted with the alternative (older) definition of moderate-to-late preterms of 33-35 wGA.

Key details from each of the individual studies can be found in Table 3.

Table 3: Key characteristics and data

Author	Country	wGA	Year	Model type*	Risk factors	Long-term morbidity	Societal costs	Industry funded	ICERs	Cost-effective	QHES score	NICE QAC
Stevens et al.[18]	USA	30-32	2000	CEA	Yes	No	No	No	US$72,712/RSVH averted	No	48	Very serious limits
Vogel et al.[10]	New Zealand	29-31	2002	CEA	No	No	Yes	Yes	NZ$98,000/RSVH averted	No	60	Potentially serious limits
Elhassan et al.[11]	USA	29-30 and 31	2006	CBA/CUA	No	Yes	Yes	No	29-30 wGA: US$675,780/QALY 31 wGA: US$1,212,497/QALY	No	67	Minor limits
Wang et al.[17]	UK	30-32[0]	2008	CUA	Yes	Yes (not in base case and no results by wGA subgroup)	Yes	No	<3 months £361,000/QALY 3-6 months £753,000/QALY 6-9 months £1,283,000/QALY	No	87	Minor limits

Wang et al.[16]	UK	30-32[0]	2011	CUA	Yes	No	No	No	≥£3,168,000/QALY	No	63	Minor limits
Salinas-Escudero et al.[14]	Mexico	29-32	2012	CEA/CUA	No	Yes	No	Yes	US$29,637/LYG<br>US$20,760/QALY	Yes	87	Minor limits
Bentley et al.[13]	UK	29-32	2013	CUA	No	Yes	No	Yes	£30,205/QALY	No	78	Minor limits
Hansen et al.[15]	USA	29-30 and 31-32	2017	CUA	No	No	No	Yes	Not reported	Yes, in infants <3 months chronological age	N/A (abstract only)	
Narayan et al.[12]	UK	29-32	2020	CUA	No	Yes	No	Yes	Base case: -£29,917/QALY<br>7 years respiratory mortality scenario: -£63,105/QALY	Yes	77	Minor limits

CBA: cost-benefit analysis; CEA: cost-effective analysis; CUA: cost-utility analysis; ICERs: incremental cost-effectiveness ratios; LYG: life-year gained; NICE: National Institute for Health and Care Excellence; QAC: quality assurance committee; QALY: quality adjusted life-year; QHES: Quality of Health Economic Studies; RSVH: respiratory syncytial virus hospitalisation; wGA: weeks' gestational age

Study quality

Overall, according to the Quality of Health Economic Studies (QHES) Instrument[19] and the National Institute of Health and Care Excellence (NICE) Quality Appraisal Checklist,[20] the included studies were mostly of moderate to good quality (see spreadsheet for individual study breakdowns). QHES scores ranged from 48 to 87 with a mean score of 70.9 (max 100). Whilst 6 of the 8 studies assessed were found to have only minor limitations, one[10] had potentially serious limitations and one[18] had very serious limitations. The two quality scales were largely well aligned, with the lowest scoring studies on the QHES scale corresponding to those identified as being more limited using the NICE checklist. The two lowest scoring studies[10,18] were the two oldest studies and deviated from a more contemporaneous format of a cost-effectiveness assessment. They were based on historical and real-life birth cohorts as opposed to decision tree analysis and calculated cost *per* RSV hospitalisation (RSVH) averted rather than QALYs.

Cost-utility analysis using a decision tree analysis was the dominant modelling approach

CUA was the most frequent type of analysis undertaken and decision tree models were used in the majority of studies. Of the nine studies reporting directly on a particular model, five reported on CUAs, two utilised CEA in isolation, and the remaining two reported on CUA alongside CEA or CBA. Similarly, six studies utilised decision tree models, the three exceptions being the historical and real-life cohort models[10,18] and a US analysis that utilised a 4-stage Markov model.[15]

All models incorporated RSVH as the primary outcome prevented by palivizumab. Other recognised outcomes including intensive care unit (ICU) admission, mortality, and long-term sequelae were accounted for in the respective studies. Indirect costs to parents varied between the studies as to whether they were considered, and which infants were affected (see below for more details). This will be an important consideration in building our model.

Included analyses were focused on high-income countries

Of the nine studies, eight were conducted in well developed, and wealthy countries:

- UK
- US
- New Zealand

Salinas-Escudero et al.[14] was the only study to assess cost-effectiveness in a less well-developed economy – Mexico – an upper-middle income country.[21]

More recent analyses were cost-effective

The publication dates ranged from 2000-2020 with studies spread evenly throughout this period. As aforementioned, the two oldest studies used cohort models,[10,18] whereas the newer studies utilised decision tree analysis/Markov model. The three studies[16-18] including risk factors were published during or before 2011 and the three studies[10,11,17] including societal costs were again older studies published during or before 2008. Interestingly, the three studies[12,14,15] which found palivizumab to be cost-effective were three of the four most recently published (during or after 2012). Notably, the study found not to be cost-effective from these four recent publications reported an ICER of £30,205/QALY and so was very close to the country-specific cost-effectiveness threshold of £30,000.[13]

Interestingly, there was a notable difference between the results of two UK studies[12,13] published seven years apart. Both models included long-term morbidity, but not risk factors or societal costs. The 2013 study[13] reported palivizumab was not cost-effective with an ICER of £30,205/QALY; however, the 2020 study[12] reported palivizumab to be dominant with an ICER of -£63,105/QALY. This emphasises the importance of the model inputs and data sources which are explored further below.

Cost-effectiveness of palivizumab

All nine studies drew a conclusion regarding the cost-effectiveness of palivizumab. As stated above, three studies[12,14,15] found palivizumab to be cost-effective for the prevention of RSV infection and six[10,11,13,16-18] found it not to be cost-effective.

Drivers of outcome varied across studies

The variables found to have the greatest influence on outcome can be summarised as follows:

- Number of palivizumab doses/cost of palivizumab

- Efficacy of palivizumab
- RSVH rates and costs
- RSV mortality
- Long term morbidity
    - Interestingly, of the four studies that included long term morbidity in their models and presented results relevant to the 29-31wGA subgroup, three suggested long-term morbidity was a key driver. In Elhassan et al.[11], reducing the quality of life with asthma from 0.89 to 0.8 resulted in ICERs less than the $200 000/QALY cost-effectiveness threshold for 29-30 wGA preterms. Moreover, sensitivity analyses from two UK models[12,13] found that the number of years accounted for in the time horizon, and cost and utilities associated with long term morbidity were amongst the top 10 most important model parameters. Specifically, when Narayan et al.[12] used 7-year data from Sigurs et al.[22,23] (as also used by Elhassan et al.[11]) in a sensitivity analysis as opposed to Shefali et al.[24] which was used by Bentley et al.[13] who assumed the effects of wheeze only lasted for the first two years of life, the cost-effectiveness of palivizumab improved markedly. However, Salinas-Escudero et al.[14] reported asthma sequelae did not affect the ICER, even over an 18-year time horizon; notably, this model used data from the Chirico G study[25] which recalls Simoes EA[26] and Ravasio R[27] studies as opposed to Sigurs et al.[22,23]

Characteristics of studies which concluded palivizumab not to be cost-effective

Of the six studies that concluded that palivizumab was not cost-effective in 29-31/2 wGA infants (or a subset thereof), four did not include long-term morbidity in the base case analysis,[10,16-18] (one included the parameter as a sensitivity analysis but this did not change the conclusion[17]), and two[11,13] included long-term morbidity in the base-case analysis. Bentley et al.[13] adopted a lifetime time horizon but assumed that the effects of wheeze only lasted for the first two years of life based on Shefali Patel et al.[24] Whereas Elhassan et al.[11] modelled the effect of asthma over an 8-year time horizon using data from Sigurs et al.[22,23]

Three studies considered risk factors within their models.[16-18] In a 2008 model, Wang et al.[17] incorporated consideration of school age siblings with conceptual age and gestational age at birth to estimate baseline hospitalisation risk, whilst in a 2011 model,

Wang et al.[16] took this a step further and considered various combinations of risk factors as to their effect on baseline hospitalisation risk. Stevens et al.[18] assessed the cost-effectiveness of palivizumab in a subgroup of 30-32 wGA infants requiring respiratory support; the incremental cost *per* hospitalisation averted reduced from $72,712 to $50,888 for this higher risk subgroup.

Across the six studies, three used the same low mortality rates which were applied to ICU admitted infants only (0.43%) from Chater et al.[28], whilst two[10,11] assumed no mortality benefit and one did not include any mortality information.[18]

Pharmaceutical costs were generally high, assuming five doses in all six studies,[10,11,13,16-18] and of the five studies with information available, four[10,16-18] assumed vial sharing/no drug wastage, whilst Elhassan et al.[11] accounted for drug wastage within the calculations. Within the sensitivity analysis carried out by Elhassan, varying the cost of palivizumab to assume no drug wastage resulted in only a very minor change to the ICER ($675,780 to $657,780 for 29-30 wGA infants). Five out of the six studies also incorporated costs associated with palivizumab administration.[10,11,13,16,17]

There was no particular trend or association to funding source within the studies which found palivizumab not to be cost-effective. One study was industry funded;[13] two were funded by a health technology association (HTA) organisation in UK;[16,17] and two did not acknowledge any funding source.[11,18]

Characteristics of studies which found palivizumab to be cost-effective

Two out of the three positive studies considered long-term morbidity in the base case of their model,[12,14] compared to only 2/6 negative studies, so potentially this plays an important role in cost-effectiveness for the 29-31 wGA subgroup. Lower palivizumab costs also appeared important. Two studies specifically reported using <5 doses (3.7 doses in Narayan et al.[12] and 4.1 doses in Salinas-Escudero et al.[14]) of palivizumab in their base case analysis, whilst the third positive study did not report the number of doses.[15] Furthermore, none of the three studies reported any palivizumab administration costs.[12,14,15] Notably, inclusion of additional risk factors or societal costs was not a pre-requisite for cost-effectiveness in these analyses, as none of the positive studies incorporated them within their model.[12,14,15]

Mortality rates were only reported/included in one of the three studies, and, as for the negative studies, the rates were very low (0.23% with palivizumab and 0.99% without palivizumab, taken from the Checchia et al.[29] meta-analysis). All three of the positive studies were also industry funded.

Key considerations for model building

When considering our own model and key questions to address, it is clear from the above that several areas require careful consideration, many of which align with the report for the 32-35 wGA subgroup.

Palivizumab dose and appropriate number of injections

It is clear that palivizumab dose and injection number are fundamental variables in any model. Therefore, consideration of how to calculate a fair and representative value will be key. The most comprehensive approach in the studies identified include calculations that take into account the variation in the likely weight of prophylaxed infants, the number of doses they will require according to their discharge date and length of the RSV season, and the number and size of vials (50mg *versus* 100mg) that this will require.[12] The decision to assume vial sharing or account for drug wastage, as well as the incorporation of administrative costs may also be crucial. Detailed examination and appraisal of these approaches will be undertaken to ensure we adopt the optimal approach.

Long term sequelae/morbidity

Of the four studies including long-term morbidity, three (75%) suggested it was a key driver in the model, and of the three positive studies, two (66%) included long-term morbidity; therefore, it is clear that inclusion of such sequelae can have marked effects on the results of the cost-effectiveness analysis. Key questions to be addressed include identification of the best dataset to estimate the duration, cost, resource use and health utilities of such long-term morbidity as these were identified as the key drivers.[11,12,13]

Palivizumab efficacy, baseline hospitalisation rate and RSV mortality

The IMpact RSV study was used by six of the eight (75%) studies reporting palivizumab efficacy rates, and whilst baseline hospitalisation rates were taken from various sources, most commonly studies included or at least incorporated the IMpact RSV study.[11,14,16,17] Other sources included country-specific cohort studies[10,18] or health insurance/pharmaceutical data on file.[13,15] Since only two sources of mortality data[28,29]

were identified in our review, consideration must be given as to whether either of these are the optimal data source to adopt in our model.

Societal costs and quality of life

All three studies including societal costs found palivizumab to not be cost-effective. However, it is still important to account for the impact of RSVH on infant and parental quality of life, time and costs alongside the direct clinical outcomes associated with prophylaxis, RSVH and subsequent morbidity. Full consideration of parental costs is required to accurately represent the burden of RSV. Our review identified a number of different approaches and data sources which could be adopted or modified for use in our model.

Model structure

The approaches to capturing and costing RSV-related outcomes adopted in the studies included in our review varied slightly, but the majority used a simple approach which followed a linear path with infection leading to RSV admission in a proportion of infants, with surviving infants then at risk of ICU admission and/or respiratory sequelae; whereas for non-hospitalised infants no further outcomes were considered.[12,13,16,17] Salinas-Escudero et al.[14] also incorporated RSV infections not causing hospitalisation which is an important area to discuss both in terms of short and potential longer term costs and utility decrements. Detailed consideration of these different approaches will be undertaken in designing our model, especially in light of non-hospitalised, medically attended RSV being a key endpoint of the trials of new preventative RSV options, such as nirsevimab.[30] This is a potentially important consideration in future cost-effectiveness assessments.

Conclusions from other systematic reviews

Four systematic reviews were identified with publication dates ranging from 2001 to 2019.[6-9] The most recently published was a Canadian systematic review by Mac et al.[6] which focussed on the cost-effectiveness of palivizumab prophylaxis in infants ranging from 29-35 wGA with or without co-morbidities and thus our review is more focussed. It should also be noted that Mac et al.[6] primarily reported on the results of published cost-effectiveness analyses, whilst our review provides a more in-depth investigation into how the models were developed (approach, assumptions, perspective(s), data source *etc*). Five of the studies in our report[10,11,13,14,17] were also identified by Mac et al.[6], but we report on an additional four studies of interest.[12,15,16,18] Although Mac et al.[6] performed a broader review across GA groups, many of their conclusions align with those drawn in our report.

Importantly, both reports state key model parameters include RSV hospitalisation rates, palivizumab efficacy, mortality rates and the number and cost of palivizumab doses.

References

[1] Paez A. Gray literature: An important resource in systematic reviews. *J Evid Based Med*. 2017;10(3):233-240.

[2] Mandrik OL, Severens JLH, Bardach A, et al. Critical Appraisal of Systematic Reviews With Costs and Cost-Effectiveness Outcomes: An ISPOR Good Practices Task Force Report. *Value Health*. 2021;24(4):463-472.

[3] McGowan J, Sampson M, Salzwedel DM, et al. PRESS Peer Review of Electronic Search Strategies: 2015 Guideline Statement. *J Clin Epidemiol*. 2016;75:40-6.

[4] Ofman JJ, Sullivan SD, Neumann PJ, et al. Examining the value and quality of health economic analyses: implications of utilizing the QHES. *J Manag Care Pharm*. 2003;9(1):53-61.

[5] Methods for the development of NICE public health guidance (third edition). Process and methods [PMG4]. Available at: https://www.nice.org.uk/process/pmg4/chapter/appendix-i-quality-appraisal-checklist-economic-evaluations. Accessed November 2021.

[6] Mac S, Sumner A, Duchesne-Belanger S, et al. Cost-effectiveness of Palivizumab for Respiratory Syncytial Virus: A Systematic Review. Pediatrics. 2019;143(5):e20184064. doi:10.1542/peds.2018-4064.

[7] Prescott WA Jr, Doloresco F, Brown J, et al. Cost-effectiveness of respiratory syncytial virus prophylaxis: a critical and systematic review. Pharmacoeconomics. 2010;28(4):279-293. doi:10.2165/11531860-000000000-00000.

[8] Andabaka T, Nickerson JW, Rojas-Reyes MX, et al. Monoclonal antibody for reducing the risk of respiratory syncytial virus infection in children. Cochrane Database of Systematic Reviews 2013, Issue 4. Art. No.: CD006602. doi:10.1002/14651858.CD006602.pub4.

[9] Simpson S, Burls A. A systematic review of the effectiveness and cost-effectiveness of palivizumab (Synagis) in the prevention of respiratory syncytial virus (RSV) infection in infants at high risk of infection. A West Midlands Development and Evaluation Service Report, 2001. ISBN: 0704423219

[10] Vogel AM, McKinlay MJ, Ashton T, et al. Cost-effectiveness of palivizumab in New Zealand. J Paediatr Child Health. 2002;38(4):352-357. doi:10.1046/j.1440-1754.2002.00790.x

[11] Elhassan NO, Sorbero ME, Hall CB, Stevens TP, Dick AW. Cost-effectiveness analysis of palivizumab in premature infants without chronic lung disease. Arch Pediatr Adolesc Med. 2006;160(10):1070-1076. doi:10.1001/archpedi.160.10.1070

[12] Narayan O, Bentley A, Mowbray K, et al. Updated cost-effectiveness analysis of palivizumab (Synagis) for the prophylaxis of respiratory syncytial virus in infant populations in the UK. J Med Econ. 2020;23(12):1640-1652. doi: 10.1080/13696998.2020.1836923.

[13] Bentley A, Filipovic I, Gooch K, et al. A cost-effectiveness analysis of respiratory syncytial virus (RSV) prophylaxis in infants in the United Kingdom. Health Econ Rev. 2013;3(1):18. doi:10.1186/2191-1991-3-18.

[14] Salinas-Escudero G, Martínez-Valverde S, Reyes-López A, et al. Cost-effectiveness analysis of the use of palivizumab in the prophylaxis of preterm patients in Mexico. Salud Publica Mex. 2012;54(1):47-59.

[15] Hansen R, McLaurin K, Sullivan S. Cost-effectiveness of palivizumab prophylaxis by gestational and chronologic age among infants at increased risk of hospitalization for respiratory syncytial virus. AMCP Managed Care and Specialty Pharmacy Annual Meeting 2017. Denver, CO United States. Journal of Managed Care and Specialty Pharmacy. 2017;23(3-A SUPPL.):S80.

[16] Wang D, Bayliss S, Meads C. Palivizumab for immunoprophylaxis of respiratory syncytial virus (RSV) bronchiolitis in high-risk infants and young children: systematic review and additional economic modelling of subgroup analyses. Health Technol Assess 2011;15(5). doi: 10.3310/hta15050.

[17] Wang D, Cummins C, Bayliss S, et al. Immunoprophylaxis against respiratory syncytial virus (RSV) with palivizumab in children: a systematic review and economic evaluation. Health Technology Assessment 2008;12: No. 36.

[18] Stevens TP, Sinkin RA, Hall CB, Maniscalco WM, McConnochie KM. Respiratory syncytial virus and premature infants born at 32 weeks' gestation or earlier: hospitalization and economic implications of prophylaxis. Arch Pediatr Adolesc Med. 2000;154(1):55-61.

[19] Ofman JJ, Sullivan SD, Neumann PJ, et al. Examining the value and quality of health economic analyses: implications of utilizing the QHES. J Manag Care Pharm. 2003;9(1):53-61.

[20] National Institute of Health and Care Excellence (NICE) Quality Appraisal Checklist. Available at https://www.nice.org.uk/process/pmg4/chapter/appendix-i-quality-appraisal-checklist-economic-evaluations. Accessed February 2022.

[21] OECD. DAC List of ODA Recipients 2021. Available at https://www.oecd.org/dac/financing-sustainable-development/development-finance-standards/DAC-List-ODA-Recipients-for-reporting-2021-flows.pdf. Accessed February 2022.

[22] Sigurs N, Bjarnason R, Sigurbergsson F, et al. Asthma and immunoglobulin E antibodies after respiratory syncytial virus bronchiolitis: a prospective cohort study with matched controls. Pediatrics. 1995;95:500–505. doi:10.1164/rccm.200406-730OC.

[23] Sigurs N, Bjarnason R, Sigurbergsson F, et al. Respiratory syncytial virus bronchiolitis in infancy is an important risk factor for asthma and allergy at age 7. Am J Respir Crit Care Med. 2000;161:1501–1507. doi:10.1164/ajrccm.161.5.9906076.

[24] Shefali-Patel D, Paris MA, Watson F, et al. RSV hospitalisation and healthcare utilisation in moderately prematurely born infants. Eur J Pediatr. 2012;171(7):1055-1061. doi: 10.1007/s00431-012-1673-0.

[25] Chirico G, Ravasio R, Sbarigia U. Cost-utility analysis of palivizumab in Italy: results from a simulation model in the prophylaxis of respiratory syncytial virus infection (RSV) among high-risk preterm infants. Italian J Pediatr 2009;35(1):4.

[26] Simoes EA, Groothuis JR, Carbonell-Estrany X, et al. Palivizumab prophylaxis, respiratory syncytial virus, and subsequent recurrent wheezing. J Pediatr 2007;151(1):34-42, e1.

[27] Ravasio R, Lucioni C, Chirico G. Costo-efficacia di palivizumab versus non profilassi nella prevenzione delle infezioni da VRS nei bambini pretermine, a diversa età gestazionale. PharmacoEconomics – Italian Research Articles 2006; 8(2):105-117.

[28] Chater T, Davey N, Draper E, et al. National Report of the Paediatric Intensive Care Audit Network 2004–2005. University of Leeds: Paediatric Intensive Care Audit Network (PICANet) 2006.

[29] Checchia PA, Nalysnyk L, Fernandes A, et al. Mortality and Morbidity Among High-Risk Preterm Infants Receiving Palivizumab Prophylaxis: A Systematic Review and Meta- Analysis. Pediatric Crit Care Med. 2011;12(5):580-588.

[30] Griffin MP, Yuan Y, Takas T, et al. Single-Dose Nirsevimab for Prevention of RSV in Preterm Infants. N Engl J Med. 2020;383(5):415-425.

The Cost-Effectiveness of Respiratory Syncytial Virus Prophylaxis in Moderate-To-Late Preterms (32-35 weeks gestational age): Summary Report of Systematic Literature Review

March 2023

Review Question

How has the health economics of palivizumab in moderate-to-late preterms been modelled and assessed?

Search methodology

Databases

The following electronic databases were searched:

- MEDLINE (via PubMed)
- EMBASE (via Ovid)
- The Cochrane Library
- CEA Registry
- Paediatric Economic Database Evaluation (PEDE) [to December 31, 2019]

In addition, other relevant studies and evidence were identified *via* bibliographies/reference lists of key articles, review of key HTA websites (NICE, SMC, CADTH *etc*) and targeted web searches for non-indexed articles, theses and dissertations, research and committee reports, government reports *etc*. (the 'grey literature').[1] To aid in identifying grey literature, the Grey Matters tool from CADTH and Opengrey will be utilised.

Restrictions

No publication period were set on the database searches.
No language limits were set on database searches, with the caveat that English translations of at least the abstract were available.

Search Dates

The time period in which the searches were executed was 15-19 November 2021.

Search Terms

The terms in Table 1 were searched in 'all fields' and combined where stated with 'Medical Subject Headings' (MeSH) in PubMed and 'Emtree Subject Headings' (ESH) in Embase:

Table 1: Search terms used in systematic review

Concept	Search Number	Search Terms	Example PubMed Results*
**Disease**	1.	RSV OR respiratory syncytial virus OR human respiratory syncytial virus [MeSH/ESH] OR bronchiolitis OR lower respiratory tract infection	69,685
**Population**	2.	((preterm OR prematur*) AND (moderate OR late)) OR ((31 OR 32 OR 33 OR 34 OR 35 OR 36) AND (gestational age OR weeks' gestational age OR wGA))	74,581
**Drug**	3.	palivizumab OR Synagis OR prophylaxis OR immunoprophylaxis OR monoclonal antibody	2,077,589
**Health Economics**	4.	cost-effectiveness OR cost analysis OR cost-utility OR cost-benefit OR economic* OR cost* OR pharmacoeconomic* OR budget* OR cost-consequence	1,458,694
	1-4 (AND)		103

The search strings were developed in line with the good practice recommendations of the International Society for Pharmacoeconomics and Outcomes Research (ISPOR) on systematic reviews with costs and cost-effectiveness[2] and assessed against the Peer Review of Electronic Search Strategies (PRESS)[3] checklist.

Inclusion and Exclusion Criteria

The condition/domain being reviewed is RSV prophylaxis in moderate-to-late preterms. The PICOS (Population, Intervention, Comparison, Outcomes and Study Design) tool was used to identify relevant references to the systematic review outcome using the inclusion and exclusion criteria in Table 2.

Table 2: PICOS framework used in systematic review

Parameter	Inclusion criteria	Exclusion criteria
**Population**	- Moderate-to-late preterms defined as 32^0-35^6 wGA who are otherwise healthy without underlying comorbidities	- Newborns born before 32^0 wGA or after 35^6 wGA<br>- Newborns with any other pathologic health condition
**Intervention/ exposure**	- Palivizumab<br>- RSV infection	- Treatment (not prophylaxis) with palivizumab for RSV infection<br>- Non-RSV based infections
**Comparator**	- No prophylaxis<br>- RSV prevention	- None
**Outcomes**<br>- **Main**<br>- **Additional**	- Model input parameters<br>- Model structure and methods, population, ICERs/results<br>- Data sources	- Any clinical efficacy and safety outcomes
**Study Design**	- Any type of health economic study published in full or abstract form<br>- Systematic reviews and meta-analyses of health economic studies	- Non-systematic review articles, letters, pre-clinical studies, case reports, expert opinions, editorials, letters, narratives, clinical trials and observational studies
**Language**	- Health economic studies in any language	- Studies without at least an English language abstract
**Publications date**	- All dates prior to literature search	- None

Data extraction

After removal of duplicates, studies were selected for inclusion in the review using a two-phase approach:

Phase 1 - the title and abstracts of potentially relevant citations identified from the electronic searches were assessed separately by two experienced reviewers to confirm relevance and inclusion in the study according to the inclusion criteria

Phase 2 - the full texts of those citations identified as relevant in phase 1 were assessed separately by two experienced reviewers to confirm relevance and inclusion in the review according to the inclusion criteria. If a consensus could not be reached on a citation, a third senior researcher made the decision.

Data was extracted from the full-text of all relevant articles identified in Phase 2 by one reviewer, and quality checked by a second reviewer. All information was inserted into an agreed Excel data extraction table template which included the following fields: Country, Population (and Perspective), Time Horizon, Key Model Assumptions, Discount Rates, Input Parameters (including direct and indirect costs, health resource utilisation, follow-up), Outcome Measures, Cost-Effectiveness Threshold, Methodology/Model Structure, Results (e.g. ICER and cost per quality-adjusted life-year) and Funding. For cost-utility models utility parameters (including quality of life tool and value set used) will also be extracted. Study investigators were not contacted for any missing/unreported data.

Quality assessment

Studies were evaluated following the good practice recommendations of ISPOR on systematic reviews with costs and cost-effectiveness.[2] Each of the studies was assigned a quality score using the Quality of Health Economic Studies (QHES) Instrument[4] and the NICE Quality Appraisal Checklist.[5]

Overview of results

Three hundred and thirty-one unique publications were identified from the systematic literature search, of which 261 were excluded based on title and abstract, and a further 44 on review of the full publication to give 26 included studies (Figure 1).

Of the included studies, five were systematic reviews[6-10] and 21 gave details of cost-effectiveness models,[11-31] with Carbonell-Estrany et al 2009[28] providing additional information on Lazaro y de Mercado et al 2006[22] which was published in Spanish. Review of the bibliographies of the systematic reviews did not identify any additional publications for inclusion.

Figure 1: PRISMA diagram

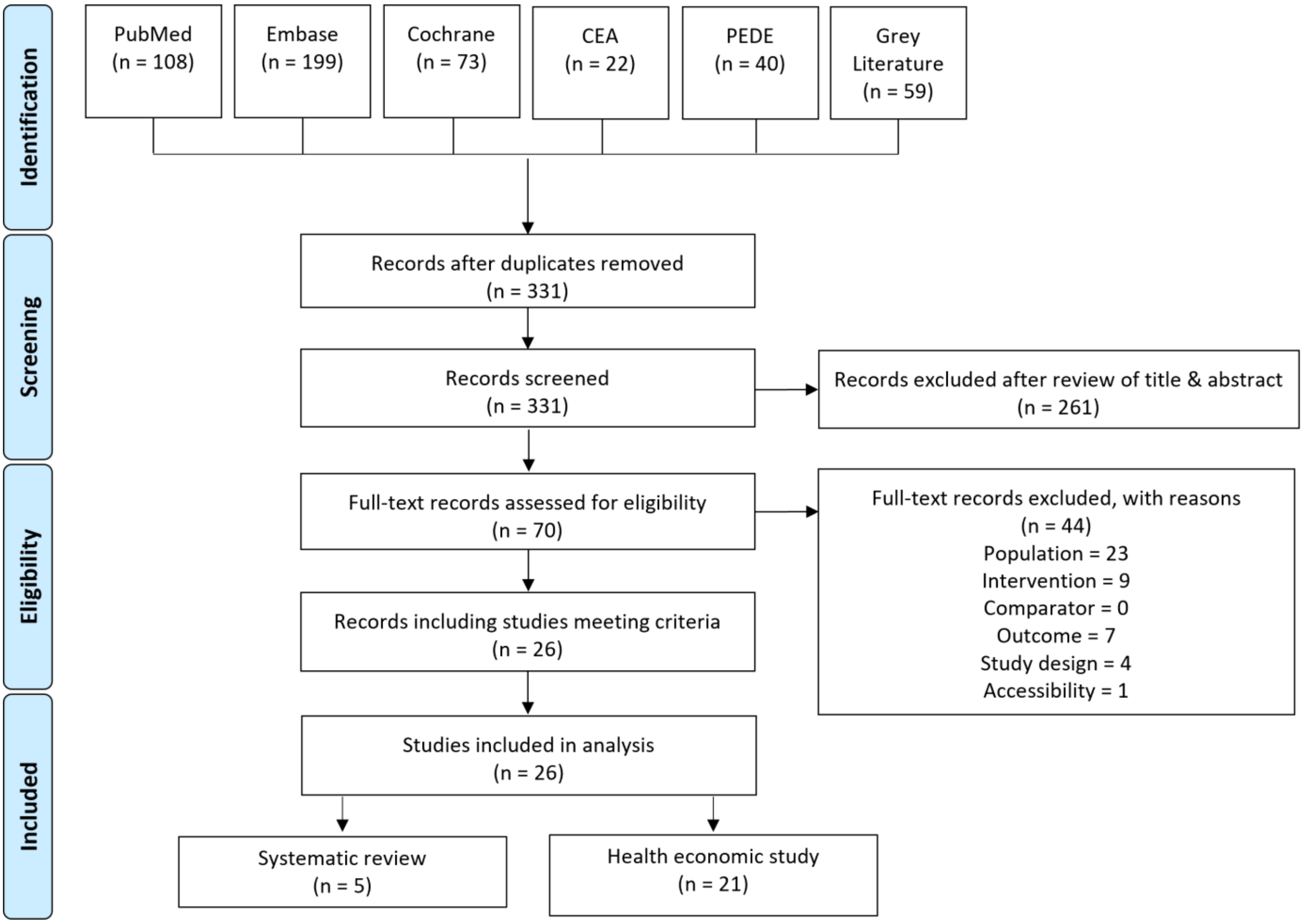

CEA: Cost-Effectiveness Analysis Registry; PEDE: Paediatric Economic Database Evaluation

Characteristics and quality of the included cost-effectiveness studies

Of the 21 publications including information on cost-effectiveness models, three were congress abstracts only,[24,25,26] three were full publications with only the abstracts available in English[22,27,29] and one was a meeting proceedings that included an overview of a non-English language publication as described above.[28] As such, data abstraction from these seven publications was limited and they were not assessed for quality as there was insufficient information available.

An overview of the 20 cost-effectiveness studies [NB two Spanish papers covered the same model[22,28]] is as follows:

- All 20 (100%) studies were conducted in seven high-income countries with 12 (60%) in Europe and eight (40%) in North America
- Studies were published between 1999 and 2020
- 18 (90%) used a decision analytic model, one (5%) used a Markov model, and one (5%) used a budget impact model (BIM)
- 12 (60%) reported a cost-utility analysis (CUA), two (10%) a cost-effectiveness analysis (CEA), five (25%) both CUA and CEA, and there was one (5%) budget impact analysis
- Nine (45%) models took a payer perspective, six (30%) a societal perspective, and five (25%) both perspectives
- Time horizon ranged from 1-year to lifetime
- 13 (65%) reported that palivizumab was cost-effective (at least in certain sub-populations)
- 13 (65%) were funded by industry

Key details from each of the individual studies can be found in Table 3.

Table 3: Key characteristics and data

Author	Country	wGA	Year	Model type*	Risk factors	Long-term morbidity	Societal costs	Industry funded	ICERs	Cost-effective	QHES score	NICE QAC
Joffe et al.[20]	USA	33-36	1999	CEA	No	No	Yes	No	Sept-Nov discharge: US$150,000/RSVH averted US$430,000/LYG Dec-Aug discharge: US$420,000/RSVH averted US$1,200,000/LYG	No	52	Potentially serious limits
Ravasio et al.[29]	Italy	33-35	2006	CEA & CUA	Not reported in abstract	Yes	No	No	€35,255.90/LYG €18,790.96/QALY	(not stated in abstract)	N/A (full paper in Italian)	
Lázaro y de Mercado et al.[22]	Spain[†, ‡]	32-35	2006	CEA & CUA	Yes	Yes	Yes	Yes	Payer: €13,849/QALY Societal: €4,605/QALY	Yes with ≥2 RF	N/A (full paper in Spanish)	
Lanctôt et al.[23]	Canada	32-35	2008	CEA & CUA	Yes	Yes	Yes	Yes	CA$20,924/QALY	Yes (with ≥2 RF or in moderate or	88	Minor limits

										high-risk categories)		
Wang et al.[31]	UK	32-34	2008	CUA	No	Yes (not in base case)	No	No	<3 months £530,000/QALY 3-6 months £954,000/QALY 6-9 months £1,922,000/QALY	No	87	Minor limits
Chirico et al.[30]	Italy	33-35	2009	CEA & CUA	No	Yes	No	No	€28,417.08 /LYG €14,937.32/QALY	Yes	61	Potentially serious limits
Smart et al.[16]	Canada	32-35	2010	CUA	Yes	Yes	Yes	No (but update of Lanctôt)	CA$30,618 to CA$31,360/QALY	Yes (with ≥2 RF or in moderate or high-risk categories)	55	Minor limits
Garcia-Altes et al.[27]	Spain[†]	32-35	2010	CEA	Not reported in abstract	No	Not reported in abstract	Not reported in abstract	Not reported in abstract	No	N/A (full paper in Catalan)	
Krilov et al.[21]	USA	32-35	2010	BIM	Yes	No	No	No	Additional cost of 9 cents *per* member assuming	Additional cost deemed acceptable	59	Very serious limits

									prophylaxed infants with 2/8 listed RFs			
Wang et al.[13]	UK	>32-34	2011	CUA	Yes	No	No	No	≥£4,320,000/QALY	No	63	Minor limits
Weiner et al.[26]	USA	32-35	2011	CUA	Yes	Yes	Yes	Yes	≥2 RF: US$3,791/QALY ≤1 RF: US$22,690/QALY	Yes (in high-risk group with ≥2 RF)	N/A (abstract only)	
Resch et al.[15]	Austria	33-35	2012	CEA & CUA	No	Yes	Yes	Yes	€35,233/LYG €24,392/QALY	Yes	82	Minor limits
Mahadevia et al.[19]	USA	32-35 and 32-34	2012	CUA	Yes	No	Yes	Yes	32-34 with 2009 AAP RF: US$44,774/QALY 32-35 with 2006 AAP RF: US$79,477/QALY 32-35 ≤1 RF: US$464,476/QALY	Yes (except 32-35 with ≤1 RF)	61	Minor limits
Weiner et al.[17]	USA	32-35 and 32-34	2012	CUA	Yes	No	Yes	Yes	32-34 with 2009 AAP RF: US$16,037/QALY 32-35 with 2006 AAP RF: US$38,244/QALY	Yes (except 32-35 with ≤1 RF)	74	Minor limits

									32-35 ≤1 RF: US$281,892/QALY			
Langenfeld et al.[25]	Netherlands	33-35	2013	CUA	Yes	No	Yes	Not available	€20,440/LYG €15,520/QALY	Yes	N/A (abstract only)	
Bentley et al.[14]	UK	33-35	2013	CUA	No	Yes	No	Yes	£99,056/QALY	No	78	Minor limits
Sanchez-Luna et al.[18]	Spain	32-35	2017	CUA	Yes	Yes	Yes	No	Payer: ICUR = €19,697.69/QALYs Societal: ICUR = €17,153.16/QALYs	Yes	81	Minor limits
Hansen et al.[24]	USA	33-34	2017	CUA	No	Yes	No	Yes	Not reported in abstract	Yes (in infants <3 months chronological age)	N/A (abstract only)	
Blanken et al.[12]	Netherlands	32-35	2018	CUA	Yes	Yes	Yes	No	€214,852/QALY	No	77	Minor limits

Narayan et al.[11]	UK	33-35	2020	CUA	No	Yes	No	Yes	Base case: -£27,657/QALY<br>7 years respiratory mortality scenario: -£58,524/QALY	Yes	77	Minor limits

† Published in Spanish; ‡ Original study carried out in 2006; additional information taken from English language summary by Carbonell-Estrany et al.[28] published in 2009 which is excluded from this table to prevent duplication. AAP: American Academy of Pediatrics; BIM: budget impact model; CEA: cost-effective analysis; CUA: cost-utility analysis; ICERs: incremental cost-effectiveness ratios; LYG: life-year gained; NICE: National Institute for Health and Care Excellence; QAC: quality assurance committee; QALY: quality adjusted life-year; QHES: Quality of Health Economic Studies; RF: risk factor; RSVH: respiratory syncytial virus hospitalisation; wGA: weeks' gestational age

Study Quality

Overall, according to the Quality of Health Economic Studies (QHES) Instrument[4] and the National Institute of Health and Care Excellence (NICE) Quality Appraisal Checklist,[5] the included studies were mostly of moderate to good quality (see spreadsheet for individual study breakdowns). QHES scores ranged from 52 to 88 with a mean score of 71.1 (max 100). Whilst 11 of the 14 studies assessed were found to have only minor limitations, two[20,30] had potentially serious limitations and one[21] had very serious limitations. The two quality scales were largely well aligned, with the lowest scoring studies on the QHES scale corresponding to those identified as being more limited using the NICE checklist. Of the three lowest scoring studies, one was an older study that did not use incremental analysis or calculate QALYs,[20] one was a BIM and therefore deviated from the typical format of a cost-effectiveness assessment,[21] and one was an update to a previous study[30] (which was itself published in Italian[29]) and so was lacking some of the detail required by the chosen instruments.

Cost-utility analysis using a decision tree analysis was the dominant modelling approach

CUA was the most frequent type of analysis undertaken and decision tree models were used in the majority of studies. Of the 20 studies reporting directly on a particular model, one study reported on a BIM,[21] two utilised CEA in isolation; 12 reported on CUAs and five reported on both CEA and CUA. Similarly, 18 studies utilised decision tree models, the two exceptions being the BIM[21] and a US analysis that utilised a 4-stage Markov model.[24] All models incorporated RSV hospitalisation (RSVH) as the primary outcome prevented by palivizumab. Consideration of other recognised outcomes including intensive care unit (ICU) admission, mortality, long-term sequelae and indirect costs to parents varied between the studies as to whether they were considered and which infants were affected (see below for more details). This will be an important consideration in building our model.

Included analyses were focussed on high-income countries

All included analyses were carried out from the perspective of wealthy, northern hemisphere countries:

- Austria
- Canada
- Italy

- The Netherlands
- Spain
- UK
- USA

Studies assessing cost-effectiveness in less developed economies were identified but did not meet the criteria for inclusion (mainly because they did not specifically assess 32-35 weeks gestational age (wGA) as a discreet sub-population).

More recent analyses were CUA and were cost-effective

The publication dates ranged from 1999-2020 with studies spread evenly throughout this period. Within the last 5-years, there have been four studies published, all of which performed a CUA.[11,12,18,24] In comparison, the four oldest studies (1999-2008) were CUA (x1), CEA (x1) or a combination of both (x2).[20,23,29,31] There was no clear trend between the newest and oldest studies in the terms of the inclusion of societal costs (new 2/4 [50%];[12,18] old 2/4 [50%][20,23]). However, a higher proportion of the more recent analyses tended to include risk factors (new 2/4 [50%];[12,18] old 1/3 [33%][23]) and long-term morbidity in the base case (new 4/4 [100%];[11,12,18,24] old 2/4 [50%][23,29]). This may have contributed to the fact that three of the four (75%)[11,18,24] most recent publications found palivizumab to be cost-effective compared to only one of the three (33%)[23] oldest studies.

Interestingly, there was a notable difference between the results of two Spanish studies[18,22] published 11 years apart. Both models included risk factors, long-term morbidity and societal costs and found palivizumab to be cost-effective; however, the 2006 study[22] reported a payer ICER of €13,849/QALY and a societal ICER €4,605/QALY whilst the 2017 study[18] reported a payer ICUR of €19,697.69/QALY and societal ICUR of €17,153.16/QALY. This emphasises the importance of the model inputs and data sources which are explored further below.

Cost-effectiveness of palivizumab

Excluding Carbonell-Estrany et al 2009[28] as it simply provides extra information on Lazaro y de Mercado et al 2006,[22] 19/20 studies drew a conclusion regarding the cost-effectiveness of palivizumab; one study written in Italian did not specifically report this in the English abstract (albeit the cost *per* QALY was <€20,000, so could be deemed cost-effective).[29] Thirteen studies found palivizumab to be cost-effective for the prevention of RSV infection[11,15-19,21-26,30] and six found it not to be cost-effective.[12,13,14,20,27,31]

Drivers of outcome varied across studies

The variables found to have the greatest influence on outcome can be summarised as follows:

- Number of palivizumab doses/cost of palivizumab
- Efficacy of palivizumab
- RSVH rates and costs
- Discriminatory power of rule used to identify high-risk infants (taken from a model which examined the cost-effectiveness of palivizumab when used in high-risk 33-35 wGA infants only)[12]
- Season length
- RSV mortality

Interestingly, inclusion of long-term morbidity *per se* was not explicitly stated to a be a major driver of cost-effectiveness despite being considered in 12 of the models. This may be attributable to the short time horizon adopted. Typically, models considered morbidity over the first two years of life based on the studies of Greenough et al 2001[32] and Shefali et al 2012.[33] Alternative data sources included the MAKI trial,[34] as used by Blanken et al[12] with a 1-year time horizon, and Simoes et al 2007,[35] as used by Chirico et al 2009,[30] with a 2-year time horizon. Of note, when longer time horizons for the impact of recurrent wheeze were considered, the effects appeared more marked. For example, when Narayan et al 2020[11] used 7-year data from Sigurs et al 1995[36] and 2000[37] in a sensitivity analysis in place of Shefali et al,[30] the cost-effectiveness of palivizumab improved markedly with the ICER/QALY falling from -£27,657 to -£58,524. Similarly, Sanchez Luna et al 2017,[18] found that adding the impact of recurrent wheeze based on a 6-year time horizon using data from Carbonell-Estrany et al 2015[38] reduced the ICUR/QALY from €19,697.69 to €17,153.16. Finally, Weiner et al 2011[26] found that a sensitivity analysis excluding recurrent wheeze consistently increased ICER/QALY in infants with ≥2 risk factors or ≤1 risk factor (increase in ICER/QALY of $3,791 to $26,290 and $22,690 to $231,784, respectively).

Characteristics of studies which concluded palivizumab not to be cost-effective

Of the six studies that concluded that palivizumab was not cost-effective in 32-35 wGA infants (or a subset thereof), four did not include long-term morbidity in the base case analysis,[13,20,27,31] (one included it as a sensitivity analysis but this did not change the conclusion[31]), and two included long-term morbidity in the base-case analysis. However,

both of the latter studies only considered the effects of wheeze on infants for a short time period. Blanken et al 2018[12] included post RSVH wheeze according to the Maki trial[34] but only followed infants for the first year of life. Bentley et al 2013[14] adopted a lifetime time horizon, but assumed that the effects of wheeze only lasted for the first two years of life based on Shefali Patel et al.[30]

Three studies considered risk factors as a means to identifying those infants at increased risk of RSVH.[12,13,31] However, only one of the studies used risk factors to identify a high-risk population in which to focus the use of palivizumab.[12] Wang et al 2008[31] incorporated consideration of school age siblings with conceptual age and gestational age at birth to estimate baseline hospitalisation risk, whilst Wang et al 2011[13] took this a step further and considered various combinations of risk factors as to their effect on baseline hospitalisation risk. The approach adopted by Wang et al resulted in very low baseline hospitalisation risk in less premature infants (included a 15% fall in RSVH rate *per* one week increase in GA at birth).[13]

Across all five studies for which information was available,[12,13,14,20,31] the mortality rates used were generally low and applied to ICU admitted infants only (0-1.2%) whilst pharmaceutical costs were generally high assuming 5 doses in 4 studies[12,13,14,31] and 4 doses using 100mg vials in the other.[20]

There was not a particular trend or association to funding source within the negative studies. One study was industry funded;[14] two were funded by a health technology association (HTA) organisation in UK;[13,31] one by a research fund;[12] and one did not acknowledge any funding source[20] (this information was not available for one study[27]).

Characteristics of studies which found palivizumab to be cost-effective

Seven out of the 13 positive studies considered long-term morbidity in the base case of their model, so it is evident that this was not a pre-requisite for cost-effectiveness. However, lower palivizumab costs did appear important. Five studies specifically reported using <4 doses of palivizumab in their base case analysis[11,15,18,21,22] and a further two used a comprehensive approach to predict patient weight and calculate a mean cost *per* milligram for palivizumab with infants born in season assumed to receive <5 doses.[17,19] Notably, only the Canadian studies by Lanctot et al 2008[23] and Smart et al 2010[16] specified >5 doses of palivizumab and did not consider long-term morbidity in the base

case analysis. However, in both cases, the models were assessing palivizumab use in high-risk populations as defined by presence of risk factors. Indeed, use of risk factors was a defining characteristic of the positive studies with 9/13 incorporating them within their model,[16-19,21,23,25,26,28] with eight studies using risk factors to define subsets of infants in which to assess cost-effectiveness.[16-19,23,25,26,28]

Mortality rates used were generally higher than those used in the negative models, with quoted rates ranging from 2.33%[39] to 8.11%.[40] The majority (10/13) of the positive studies were also industry funded.[11,15,17,19,22-26,30]

Key considerations for model building

Considering our own model and key questions to address and resolve, it is clear from the above that several areas require careful consideration.

Palivizumab dose and appropriate number of injections

It is clear that palivizumab dose and injection number are fundamental variables in any model. Therefore, consideration of how to calculate a fair and representative value will be key. The most comprehensive approaches in the studies identified include calculations that take account of the variation in the likely weight of prophylaxed infants, the number of doses they will require according to their discharge date and length of the RSV season, and the number and size of vials that this will require.[11,17,21] Detailed examination and appraisal of these approaches will be undertaken to ensure we adopt the optimal approach.

Long term sequelae/morbidity

Although not identified as a major driver of outcome by study authors, at least in the short term, it is clear that accounting for long-term morbidity can have marked effects on the results of any analysis.[11,18,26] Key questions to be addressed include identification of the best dataset to estimate the impact (wheeze *vs* asthma; all infants *vs* ICU admitted infants) and duration (throughout childhood? Into adulthood?) of such morbidity not only in terms of healthcare utilisation that accrues, but also its impact on the quality of life of the affected infants and potentially parents.

Baseline hospitalisation rate and the impact of risk factors

Baseline hospitalisation rates were taken from various sources including the IMpact RSV study,[41] country-specific databases/studies and in some more recent studies, the published sub-analyses of the IMpact RSV study.[42] Similarly, risk factors were used in

various combinations and methodologies to either define specific high-risk groups or modify the risk of hospitalisation for the age group as a whole. We will be utilising the International Risk Scoring Tool within our model.[43]

Model structure and incorporation of ICU admission, long-term sequelae *etc*

Various approaches to capturing and costing RSV-related outcomes were adopted in the studies included in our review. A fundamental question will therefore be which of these approaches we follow/build upon. The simplest approaches identified followed a linear path with infection leading to RSV admission in a proportion of infants, with surviving infants then at risk or respiratory sequelae; whereas for non-hospitalised infants no further outcomes were considered.[14,31] In contrast, in the most complex models, every outcome is considered for every infant, albeit at a different rate and/or degree of severity.[18,30] RSV infections not causing hospitalisation are an important area to discuss both in terms of short and potential longer term costs and utility decrements. Detailed consideration of these different approaches will be undertaken in designing our model.

Other considerations

Alongside the direct clinical outcomes associated with RSVH and subsequent morbidity, it will also be important to account for the impact on infant and parental quality of life. Similarly, parental time and costs should also be accounted for during an infant's RSVH course. Our review identified a number of approaches and data sources which could be adopted or modified for use in our model.

Conclusions from other systematic reviews

Five systematic reviews were identified with publication dates ranging from 2001 to 2019.[6-10] The most recently published was a Canadian systematic review by Mac et al.[6] which focussed on the cost-effectiveness of palivizumab prophylaxis in infants ranging from 29-35 wGA with or without co-morbidities and thus our review is more focussed. It should also be noted that Mac et al.[6] primarily reported on the results of published cost-effectiveness analyses, whilst our review provides a more in-depth investigation into how the models were developed (approach, assumptions, perspective(s), data source *etc*). Nine of the studies[12,14-19,30,31] in our report were also identified by Mac et al.[6], but we report on an additional 12 studies of interest.[11,13,20-29] Although Mac et al.[6] performed a broader review across GA groups, many of their conclusions align with those drawn in our report. Importantly, both reports state key model parameters should include RSV hospitalisation rates, palivizumab efficacy, mortality rates and the number and cost of palivizumab doses.

References

[1] Paez A. Gray literature: An important resource in systematic reviews. *J Evid Based Med*. 2017;10(3):233-240.

[2] Mandrik OL, Severens JLH, Bardach A, et al. Critical Appraisal of Systematic Reviews With Costs and Cost-Effectiveness Outcomes: An ISPOR Good Practices Task Force Report. *Value Health*. 2021;24(4):463-472.

[3] McGowan J, Sampson M, Salzwedel DM, et al. PRESS Peer Review of Electronic Search Strategies: 2015 Guideline Statement. *J Clin Epidemiol*. 2016;75:40-6.

[4] Ofman JJ, Sullivan SD, Neumann PJ, et al. Examining the value and quality of health economic analyses: implications of utilizing the QHES. *J Manag Care Pharm*. 2003;9(1):53-61.

[5] Methods for the development of NICE public health guidance (third edition). Process and methods [PMG4]. Available at: https://www.nice.org.uk/process/pmg4/chapter/appendix-i-quality-appraisal-checklist-economic-evaluations. Accessed November 2021.

[6] Mac S, Sumner A, Duchesne-Belanger S, et al. Cost-effectiveness of Palivizumab for Respiratory Syncytial Virus: A Systematic Review. Pediatrics. 2019;143(5):e20184064. doi:10.1542/peds.2018-4064.

[7] Prescott WA Jr, Doloresco F, Brown J, et al. Cost effectiveness of respiratory syncytial virus prophylaxis: a critical and systematic review. Pharmacoeconomics. 2010;28(4):279-93. doi:10.2165/11531860-000000000-00000.

[8] Blanken M, Bont L, Rovers M. The cost-effectiveness of palivizumab in the prevention of respiratory syncytial virus bronchiolitis: A systematic review. Current Respiratory Medicine Reviews. 2011;7(3):203-212. doi:10.2174/157339811795589531.

[9] Simpson S, Burls A. A systematic review of the effectiveness and cost-effectiveness of palivizumab (Synagis) in the prevention of respiratory syncytial virus (RSV) infection in infants at high risk of infection. A West Midlands Development and Evaluation Service Report, 2001. ISBN: 0704423219

[10] Andabaka T, Nickerson JW, Rojas-Reyes MX, et al. Monoclonal antibody for reducing the risk of respiratory syncytial virus infection in children. Cochrane Database of Systematic Reviews 2013, Issue 4. Art. No.: CD006602. doi:10.1002/14651858.CD006602.pub4.

[11] Narayan O, Bentley A, Mowbray K, et al. Updated cost-effectiveness analysis of palivizumab (Synagis) for the prophylaxis of respiratory syncytial virus in infant populations in the UK. J Med Econ. 2020;23(12):1640-1652. doi: 10.1080/13696998.2020.1836923.

[12] Blanken MO, Frederix GW, Nibbelke EE, et al. Cost-effectiveness of rule-based immunoprophylaxis against respiratory syncytial virus infections in preterm infants. Eur J Pediatr. 2018;177:133–144. doi:10.1007/s00431-017-3046-1.

[13] Wang D, Bayliss S, Meads C. Palivizumab for immunoprophylaxis of respiratory syncytial virus (RSV) bronchiolitis in high-risk infants and young children: systematic review and additional economic modelling of subgroup analyses. Health Technol Assess 2011;15(5).

[14] Bentley A, Filipovic I, Gooch K, et al. A cost-effectiveness analysis of respiratory syncytial virus (RSV) prophylaxis in infants in the United Kingdom. Health Econ Rev. 2013;3(1):18. doi:10.1186/2191-1991-3-18.

[15] Resch B, Sommer C, Nuijten MJ, et al. Cost-effectiveness of Palivizumab for Respiratory Syncytial Virus Infection in High-risk Children, Based on Long-term Epidemiologic Data From Austria. Pediatr Infect Dis J 2012;31: e1–e8. doi:10.1097/INF.0b013e318235455b

[16] Smart KA, Paes BA, Lanctôt KL. Changing costs and the impact on RSV prophylaxis. J Med Econ. 2010;13(4):705-8. doi:10.3111/13696998.2010.535577.

[17] Weiner LB, Masaquel AS, Polak MJ, et al. Cost-effectiveness analysis of palivizumab among pre-term infant populations covered by Medicaid in the United States. J Med Econ. 2012;15(5):997-1018. doi:10.3111/13696998.2012.672942.

[18] Sanchez-Luna M, Burgos-Pol R, Oyagüez I, et al. Cost-utility analysis of Palivizumab for Respiratory Syncytial Virus infection prophylaxis in preterm infants: update based on the clinical evidence in Spain. BMC Infect Dis. 2017;17(1):687. doi:10.1186/s12879-017-2803-0.

[19] Mahadevia PJ, Masaquel AS, Polak MJ, et al. Cost utility of palivizumab prophylaxis among pre-term infants in the United States: a national policy perspective. J Med Econ 2012;15:987–96. doi:10.3111/13696998.2012.690013.

[20] Joffe S, Ray GT, Escobar GJ, et al. Cost-effectiveness of respiratory syncytial virus prophylaxis among preterm infants. Pediatrics. 1999;104(3 Pt 1):419-27. doi:10.1542/peds.104.3.419.

[21] Krilov LR, Palazzi DL, Fernandes AW, et al. Prevalence of respiratory syncytial virus (RSV) risk factors and cost implications of immunoprophylaxis to infants 32 to 35 weeks gestation for health plans in the United States. Value Health. 2010;13(1):77-86. doi:10.1111/j.1524-4733.2009.00586.x.

[22] Lázaro y de Mercado P, Figueras Aloy J, Doménech Martínez E, et al. The efficiency (cost-effectiveness) of palivizumab as prophylaxis against respiratory syncytial virus infection in premature infants with a gestational age of 32-35 weeks in Spain. An Pediatr (Barc). 2006;65(4):316-24. doi:10.1157/13092505.

[23] Lanctôt KL, Masoud ST, Paes BA, et al. The cost-effectiveness of palivizumab for respiratory syncytial virus prophylaxis in premature infants with a gestational age of 32-35 weeks: a Canadian-based analysis. Curr Med Res Opin. 2008;24(11):3223-37. doi:10.1185/03007990802484234.

[24] Hansen R, McLaurin K, Sullivan S. Cost-effectiveness of palivizumab prophylaxis by gestational and chronologic age among infants at increased risk of hospitalization for respiratory syncytial

virus. AMCP Managed Care and Specialty Pharmacy Annual Meeting 2017. Denver, CO United States. Journal of Managed Care and Specialty Pharmacy. 2017;23(3-A SUPPL.):S80.

25 Langenfeld MK, Visser S. Cost-effectiveness analysis of palivizumab as a prophylaxis for respiratory syncytial virus (RSV) infection in high-risk late preterm infants in the Netherlands. ISPOR 16th Annual European Congress. Dublin, Ireland, 2013. Value in Health. 2013;16(7):A372.

26 Weiner LB, Polak MJ, Masaquel A, et al. Cost-effectiveness of respiratory syncytial virus prophylaxis with palivizumab among preterm infants covered by medicaid in the United States. 16th Annual International Meeting of the International Society for Pharmacoeconomics and Outcomes Research, ISPOR 2011. Baltimore, United States. Value in Health. 2011;14(3):A118.

27 Garcia-Altes A, Paladio N, Tebe C, et al. Cost-effectiveness analysis of the administration of palivizumab as prophylaxis of severe bronchiolitis due to respiratory syncytial virus. Pediatria Catalana. 2010;70(2):57-64.

28 Carbonell-Estrany X, Lázaro y de Mercado P. Health economics and RSV. Paediatric Respiratory Reviews. 10(SUPPL. 1) (pp 12-13), 2009. doi:10.1016/S1526-0542%2809%2970006-5

29 Ravasio R, Lucioni C, Chirico G. Cost-effectiveness analysis of palivizumab versus no prophylaxis in the prevention of respiratory syncytial virus infections among premature infants, with different gestational ages. PharmacoEconomics - Italian Research Articles. 2006;8(2) :105-117. doi: 10.1007/bf03320561.

30 Chirico G, Ravasio R; Sbarigia U. Cost-utility analysis of palivizumab in Italy: results from a simulation model in the prophylaxis of respiratory syncytial virus infection (RSV) among high-risk preterm infants. Italian Journal of Pediatrics. 2009;35:4. doi:10.1186/1824-7288-35-4.

31 Wang D, Cummins C, Bayliss S, et al. Immunoprophylaxis against respiratory syncytial virus (RSV) with palivizumab in children: a systematic review and economic evaluation. Health Technology Assessment 2008;12: No. 36.

32 Greenough A, Cox S, Alexander J, et al. Health care utilisation of infants with chronic lung disease, related to hospitalisation for RSV infection. Arch Dis Child. 2001;85(6):463-468. doi: 10.1136/adc.85.6.463.

33 Shefali-Patel D, Paris MA, Watson F, et al. RSV hospitalisation and healthcare utilisation in moderately prematurely born infants. Eur J Pediatr. 2012;171(7):1055-1061. doi: 10.1007/s00431-012-1673-0.

34 Blanken MO, Rovers MM, Molenaar JM, et al. Respiratory Syncytial Virus and Recurrent Wheeze in Healthy Preterm Infants. N Engl J Med. 2013; 368:1791-1799. doi: 10.1056/NEJMoa1211917.

35 Simoes EA, Groothuis JR, Carbonell-Estrany X, et al. Palivizumab prophylaxis, respiratory syncytial virus, and subsequent recurrent wheezing. J Pediatr. 2007;151(1):34-42. doi:10.1016/j.jpeds.2007.02.032.

[36] Sigurs N, Bjarnason R, Sigurbergsson F, et al. Asthma and immunoglobulin E antibodies after respiratory syncytial virus bronchiolitis: a prospective cohort study with matched controls. Pediatrics. 1995;95:500–505. doi:10.1164/rccm.200406-730OC.

[37] Sigurs N, Bjarnason R, Sigurbergsson F, et al. Respiratory syncytial virus bronchiolitis in infancy is an important risk factor for asthma and allergy at age 7. Am J Respir Crit Care Med. 2000;161:1501–1507. doi:10.1164/ajrccm.161.5.9906076.

[38] Carbonell-Estrany X, Pérez-Yarza EG, García LS, et al. Long-term burden and respiratory effects of respiratory Syncytial virus hospitalization in preterm infants-the SPRING study. PLoS One. 2015;8:10(5). doi:10.1371/journal.pone.0125422

[39] Sanchez-Luna M, Elola FJ, Fernandez-Perez C, et al. (2016): Trends in respiratory syncytial virus bronchiolitis hospitalizations in children less than 1 year: 2004–2012. Curr Med Res Opin. 2016;32(4):693-8. doi:10.1185/03007995.2015.1136606.

[40] Sampalis JS. Morbidity and mortality after RSV-associated hospitalizations among premature Canadian infants. J Pediatr. 2003 Nov;143(5 Suppl):S150-6. doi:10.1067/s0022-3476(03)00513-4.

[41] IMpact RSV Study Group. Palivizumab, a humanized respiratory syncytial virus monoclonal antibody, reduces hospitalization from respiratory syncytial virus infection in high-risk infants. Pediatrics. 1998;102(3):531-7.

[42] Notario G, Vo P, Gooch K, et al. Respiratory syncytial virus-related hospitalization in premature infants without bronchopulmonary dysplasia: subgroup efficacy analysis of the IMpact-RSV trial by gestational age group. Pediatr Health Med Ther. 2014;5:43–48.

[43] Blanken MO, Paes B, Anderson EJ, et al. Risk scoring tool to predict respiratory syncytial virus hospitalisation in premature infants. Pediatr Pulmonol. 2018;53(5):605-612. doi: 10.1002/ppul.23960.
