# Supplementary material for: A new cost-utility analysis assessing risk factor-guided prophylaxis with palivizumab for the prevention of severe respiratory syncytial virus infection in Italian infants born at 29–35 weeks’ gestational age
Source: PLoS One. 2023 Aug 10;18(8):e0289828. doi: 10.1371/journal.pone.0289828 (PMC10414677; doi:10.1371/journal.pone.0289828)
Supplement: S1 Table — (PDF) [file pone.0289828.s002.pdf]

Table S1 Source data and predictive accuracy of the International Risk Scoring Tool[1]

**Source data**	**AUROC (Sensitivity; Specificity)**
- *Development* (N=13475): PICNIC (Canada); FLIP-2 (Spain); RISK (Netherlands); REPORT (USA); IBC (Italy); PONI (Europe, Central America, Asia & Middle East)<br>- *Validation* (N=1078): PREMI (Ireland)	0.773<br>(68.9%; 73.0%)

AUROC: area under the receiver operating characteristic curve, where 1 equals perfect predictive accuracy; FLIP: Risk Factors Linked to RSV Infection Requiring Hospitalization in Premature Infants study; IBC: Italian Birth Cohort; PICNIC: Pediatric Investigators Collaborative Network on Infections in Canada study; PONI: Predictors Associated with RSV Hospitalization in Nonprophylaxed, Premature Infants study; PREMI: Preterm Risk Estimation Measure for RSVH in Ireland study; REPORT: Respiratory Events Among Preterm Infants Outcomes and Risk Tracking study; RISK: RISK study; RSVH: RSV-related hospitalization; RST: risk scoring tool; sensitivity: proportion of RSVHs correctly identified; specificity: proportion of non-RSVHs correctly identified

References

1 Blanken MO, Paes B, Anderson EJ, Lanari M, Sheridan-Pereira M, Buchan S, et al. Risk scoring tool to predict respiratory syncytial virus hospitalisation in premature infants. Pediatr Pulmonol. 2018;53(5):605–12. https://doi.org/10.1002/ppul.23960.
