# Supplementary material for: A new cost-utility analysis assessing risk factor-guided prophylaxis with palivizumab for the prevention of severe respiratory syncytial virus infection in Italian infants born at 29–35 weeks’ gestational age
Source: PLoS One. 2023 Aug 10;18(8):e0289828. doi: 10.1371/journal.pone.0289828 (PMC10414677; doi:10.1371/journal.pone.0289828)
Supplement: S2 Table — (PDF) [file pone.0289828.s003.pdf]

Table S2 Risk groups in the International Risk Scoring Tool

RST	Risk Score	Proportion of infants in risk group[1,*]	RSVH rate (%)
**IRST[1]**	**Low: ≤19**	79.4%	1.0%
	**Moderate: 20-45**	19.9%	3.3%
	**High: 50-56**	0.7%	9.5%

*Using pooled dataset of infants born 33–35 weeks' gestational age with complete data for all risk factors. RSV: respiratory syncytial virus; RSVH: RSV-related hospitalization

References

1 Paes B, Fullarton JR, Rodgers-Gray BS, Carbonell-Estrany X. Adoption in Canada of an international risk scoring tool to predict respiratory syncytial virus hospitalization in moderate-to-late preterm infants. *Curr Med Res Opin*. 2021;37(7):1149-1153.
