# Supplementary material for: A new cost-utility analysis assessing risk factor-guided prophylaxis with palivizumab for the prevention of severe respiratory syncytial virus infection in Italian infants born at 29–35 weeks’ gestational age
Source: PLoS One. 2023 Aug 10;18(8):e0289828. doi: 10.1371/journal.pone.0289828 (PMC10414677; doi:10.1371/journal.pone.0289828)
Supplement: S3 Table — (PDF) [file pone.0289828.s004.pdf]

Table S3 Rates of long-term respiratory morbidity

Year	Palivizumab		No Palivizumab		Source(s)
	RSVH	No RSVH	RSVH	No RSVH	
**0-1**	18.4%	5.4%	41.4%	12.1%	Respiratory morbidity from SPRING study (Carbonell-Estrany *et al*. 2015[1]), as modified by Sanchez Luna *et al*. 2017[2] to impose palivizumab efficacy using data from Blanken *et al* 2013[3], Simoes *et al*. 2007[4] & Yoshihara *et al*. 2013[5]
**1-2**	18.4%	5.4%	41.4%	12.1%	
**2-3**	11.1%	5.8%	29.3%	15.4%	
**3-4**	6.1%	4.2%	18.6%	12.6%	
**4-5**	4.4%	2.7%	15.0%	9.3%	
**5-6**	3.3%	2.5%	12.4%	9.7%	
**6-7**	2.9%	2.3%	12.4%	9.7%	Sigurs *et al*. 2000[6] adjusted to fit SPRING[1] and modified as above
**7-13**	2.3%	1.5%	17.4%	11.0%	Sigurs *et al*. 2005[7] adjusted to fit SPRING[1] and modified as above
**13-18**	1.8%	1.2%	22.4%	14.7%	Sigurs *et al*. 2010[8] adjusted to fit SPRING[1] and modified as above

RSV: respiratory syncytial virus; RSVH: RSV-related hospitalization

For infants with RSVH, long-term respiratory morbidity rates for up to 18 years are as displayed in Table S3. For infants with medically-attended RSV infection (MARI), the corresponding rates were taken from those labelled 'No RSVH' in Table S3. Infants without an RSV infection or an RSV infection not requiring any medical management were assumed to have some background respiratory morbidity for up to 6 years, using the same rates as for MARI.

References

1 Carbonell-Estrany X, Pérez-Yarza EG, Sanchez García L, et al. Long-Term Burden and Respiratory Effects of Respiratory Syncytial Virus Hospitalization in Preterm Infants-The SPRING Study. *PLoS One* 2015;10(5):e0125422.

2 Sanchez-Luna M, Burgos-Pol R, Oyagüez I, et al. Cost-utility analysis of Palivizumab for Respiratory Syncytial Virus infection prophylaxis in preterm infants: update based on the clinical evidence in Spain. *BMC Infect Dis*. 2017;17(1):687.

3 Blanken MO, Rovers MM, Molenaar JM, et al. Respiratory syncytial virus and recurrent wheeze in healthy preterm infants. N Engl J Med 2013;368(19):1791–1799.

4 Simoes E, Groothuis JR, Carbonell-Estrany X, et al. Palivizumab prophylaxis, respiratory syncytial virus, and subsequent recurrent wheezing. *J Pediatr*. 2007;151(1):34–42.

5 Yoshihara S, Kusuda S, Mochizuki H, et al. Effect of palivizumab prophylaxis on subsequent recurrent wheezing in preterm infants. Pediatrics 2013;132(5):811–818.

6 Sigurs N, Bjarnason R, Sigurbergsson F, Kjellman B. Respiratory syncytial virus bronchiolitis in infancy is an important risk factor for asthma and allergy at age 7. *Am J Respir Crit Care Med*. 2000;161(5):1501-1507.

7 Sigurs N, Gustafsson PM, Bjarnason R, et al. Severe respiratory syncytial virus bronchiolitis in infancy and asthma and allergy at age 13. Am J Respir Crit Care Med 2005;171(2):137-141.

8 Sigurs N, Aljassim F, Kjellman B, et al. Asthma and allergy patterns over 18 years after severe RSV bronchiolitis in the first year of life. *Thorax*. 2010;65(12):1045–1052.
