# Supplementary material for: A new cost-utility analysis assessing risk factor-guided prophylaxis with palivizumab for the prevention of severe respiratory syncytial virus infection in Italian infants born at 29–35 weeks’ gestational age
Source: PLoS One. 2023 Aug 10;18(8):e0289828. doi: 10.1371/journal.pone.0289828 (PMC10414677; doi:10.1371/journal.pone.0289828)
Supplement: S4 Table — (PDF) [file pone.0289828.s005.pdf]

Table S4 Probability distributions used in probabilistic sensitivity analysis

Parameter	Point estimate	Distribution	Measure of dispersion	Dispersion values	Source
Annual cost of respiratory sequelae	€1535.90	Gamma	Max, Min	1228.72, 1843.08	1
Average birthweight 29-31wGA	1,382.98g	Normal	Mean, SD	1382.98, 138.30	2
Average birthweight 32-35wGA	2,203.43g	Normal	Mean, SD	2203.43, 220.34	2
Background utility score	0.95	Beta	Max, Min	1.00, 0.76	3
Clinician consultation	€20.66	Gamma	Max, Min	24.79, 16.53	4
Cost Childcare RSVH1	€58.30	Gamma	Max, Min	69.96, 46.64	5
Cost Childcare RSVH2	€58.30	Gamma	Max, Min	69.96, 46.64	5
Cost MARI missed work	€20.25	Gamma	Max, Min	24.30, 16.20	6
Cost MARI transport	€15.34	Gamma	Max, Min	18.41, 12.27	7
Cost missed work RSVH1	€577.80	Gamma	Max, Min	693.36, 462.24	5,6
Cost missed work RSVH2	€577.80	Gamma	Max, Min	693.36, 462.24	5,6
Cost OOPs RSVH1	€171.72	Gamma	Max, Min	206.06, 137.38	5
Cost OOPs RSVH2	€171.72	Gamma	Max, Min	206.06, 137.38	5
Cost per 100mg vial	€814.34	Gamma	Max, Min	977.21, 651.47	8
Cost per 50mg vial	€490.37	Gamma	Max, Min	588.44, 392.30	8
Cost transport RSVH1	€62.73	Gamma	Max, Min	75.28, 50.18	5
Cost transport RSVH2	€62.73	Gamma	Max, Min	75.28, 50.18	5
Cost transport to injections 29-31wGA	€64.58	Gamma	Max, Min	77.49, 51.66	7
Cost transport to injections 32-35wGA	€64.58	Gamma	Max, Min	77.49, 51.66	7
Discount rate costs	3.00%	Normal	Mean, SD		N/A
Discount rate utilities	3.00%	Normal	Mean, SD		N/A
Disutility whilst in hospital	0.60	Beta	Max, Min	0.72, 0.48	9,10
ED visit cost	€241.05	Gamma	Max, Min	289.26, 192.84	11
Hospital length of stay 29-31wGA	6.80 days	Normal	Mean, SD	6.80, 0.68	Manzoni P, Personal Communication
Hospital length of stay 32-35wGA	6.80 days	Normal	Mean, SD	6.80, 0.68	Manzoni P, Personal Communication
Hospitalization cost bundle	€5,768.00	Gamma	Max, Min	6921.60, 4614.40	4
ICU cost per day	€11179.00	Gamma	Max, Min	13414.80, 8943.20	4
ICU rate PVZ 29-31wGA	8.70%	Beta	Max, Min	10.44%, 6.96%	Calculated from 12,13
ICU rate PVZ 32-35wGA	8.70%	Beta	Max, Min	10.44%, 6.96%	Calculated from 12,13
LT morbidity rates no PVZ after RSV (multiplier)	1.00	Beta	Max, Min	-	1,3,4,5,6,7, 8,14

LT morbidity rates no PVZ no RSV (multiplier)	1.00	Beta	Max, Min	-	1,3,4,5,6,7,8,14
LT morbidity rates PVZ after RSV (multiplier)	1.00	Beta	Max, Min	-	1,3,4,5,6,7,8,14
LT morbidity rates PVZ no RSV (multiplier)	1.00	Beta	Max, Min	-	1,3,4,5,6,7,8,14
MARI rates PVZ ED only 29-31wGA	4.26%	Beta	Max, Min	5.11%, 3.41%	14
MARI rates PVZ ED only 32-35wGA	2.95%	Beta	Max, Min	3.54%, 2.36%	14
Mean number of injections given 29-31wGA	4.21	Normal	Mean, SD	4.21, 0.42	Manzoni P, Personal Communication
Mean number of injections given 32-35wGA	4.21	Normal	Mean, SD	4.21, 0.42	Manzoni P, Personal Communication
Cost of missed work for injections 29-31wGA	€85.25	Gamma	Max, Min	102.30, 68.20	6
Cost of missed work for 32-35wGA	€85.25	Gamma	Max, Min	102.30, 68.20	6
Mortality	0.43%	Normal	Mean, SD	0.43%, 0.04%	15,16
Non-prophylaxed first hospitalization rate 29-31wGA	5.88%	Beta	Max, Min	7.06%, 4.70%	17
Non-prophylaxed first hospitalization rate 32-35wGA	6.30%	Beta	Max, Min	7.56%, 5.04%	1
Non-prophylaxed ICU rate 29-31wGA	20.00%	Beta	Max, Min	24.00%, 16.00%	Calculated from 12,13
Non-prophylaxed ICU rate 32-35wGA	20.00%	Beta	Max, Min	24.00%, 16.00%	Calculated from 12,13
Non-prophylaxed second hospitalization rate 29-31wGA	2.47%	Beta	Max, Min	2.96%, 1.98%	18
Non-prophylaxed second hospitalization rate 32-35wGA	4.21%†	Beta	Max, Min	5.05%, 3.36%	18,19
Palivizumab relative risk reduction 29-31wGA	0.63	Normal	Mean, SD	0.63, 0.06	20
Palivizumab relative risk reduction 32-35wGA	0.82	Normal	Mean, SD	0.82, 0.08	20
RSV hospitalized utility score	0.88	Beta	Max, Min	1.00, 0.70	3
Subsequent RSV rate PVZ 29-31wGA	2.47%	Beta	Max, Min	2.96%, 1.98%	18
Subsequent RSV rate PVZ 32-35wGA	4.21%†	Beta	Max, Min	5.05%, 3.36%	18,19
Utility with significant respiratory sequelae	0.79	Beta	Max, Min	0.95, 0.63	21

†In base case, calculated proportionally from risk of first RSVH in high- and moderate-risk (using IRST) adjusted for relative risk of 1st and 2nd RSVH in overall population 3.70% *vs* 2.47%, respectively. ED: Emergency Department; ICU: intensive care unit; LOS: length of stay; MARI: medically-attended RSV infection; OOP: out of pocket (expenses); RRR: relative risk reduction; RSV: respiratory syncytial virus; RSVH: RSV-related hospitalization; wGA: weeks' gestational age

References

1 Dal Negro RW, Distante C, Bonadiman L, Turco P, Iannazzo S. Cost of persistent asthma in Italy. Multidiscip Respir Med. 2016;11:44. https://doi.org/doi:10.1186/s40248-016-0080-1.

2 Bellù R, Di Lallo D, Fabianao A, Franco F, Gagliardi L, Turoli D **(eds.)**. Italian Neonatal Network INNSIN Anno 2020. Italian Neonatal Network. 2020.

[3] Greenough A, Alexander J, Burgess S, Bytham J, Chetcuti PA, Hagan J, et al. Health care utilisation of prematurely born, preschool children related to hospitalisation for RSV infection. Arch Dis Child. 2004;89(7):673–78. https://doi.org/10.1136/adc.2003.036129.

[4] Italian Ministry of Health. Remunerazione delle prestazioni di assistenzaospedaliera per acuti, assistenza ospedaliera di riabilitazione e di lungodegenza post acuzie e di assistenza specialistica ambulatoriale. https://www.gazzettaufficiale.it/eli/gu/2013/01/28/23/so/8/sg/pdf. Accessed January 2023.

[5] Mitchell I, Defoy I, Grubb E. Burden of Respiratory Syncytial Virus Hospitalizations in Canada. Can Respir J. 2017;2017:4521302. https://doi.org/doi:10.1155/2017/4521302.

[6] Italian National Institute of Statistics. Rapporto Annualle 2022. La situazione del Paese. https://www.istat.it/storage/rapporto-annuale/2022/Rapporto_Annuale_2022.pdf. Accessed January 2023.

[7] Automobile Club of Italy. Kilometer costs. https://www.aci.it/i-servizi/servizi-online/costi-chilometrici.html. Accessed January 2023.

[8] [Region of Veneto Regional Council]. Linee di indirizzo per l'impiego del medicinale Synagis® (palivizumab) nella Regione del Veneto. Available at: https://bur.regione.veneto.it/BurvServices/pubblica/Download.aspx?name=4_Allegato_DDR_4_12-01-2016_316357.pdf&type=7&storico=False. Accessed January 2023.

[9] Weiner LB, Masaquel AS, Polak MJ, Mahadevia PJ. Cost-effectiveness analysis of palivizumab among pre-term infant populations covered by Medicaid in the United States. J Med Econ. 2012;15(5):997–1018. https://doi.org/10.3111/13696998.2012.672942.

[10] Leidy NK, Margolis MK, Marcin JP, Flynn JA, Frankel LR, Johnson S, et al. The impact of severe respiratory syncytial virus on the child, caregiver, and family during hospitalization and recovery. Pediatrics. 2005;115(6):1536–46. https://doi.org/10.1542/peds.2004-1149.

[11] Italian Ministry of Health. The Mattoni Project. Proposta metodologica per la valutazione dei costi dell'emergenza. 23 January 2007.

[12] Ravasio R, Lucioni C, Chirico G. Costo-efficacia di palivizumab versus non profilassi nella prevenzione delle infezioni da VRS nei bambini pretermine, a diversa età gestazionale. PharmacoEconomics – Italian Research Articles. 2006;8:105–117. https://doi.org/10.1007/BF03320561

[13] Cutrera R, Wolfler A, Picone S, Rossi GA, Gualberti G, Merolla R, et al. Impact of the 2014 American Academy of Pediatrics recommendation and of the resulting limited financial coverage by the Italian Medicines Agency for palivizumab prophylaxis on the RSV-associated hospitalizations in preterm infants during the 2016-2017 epidemic season: a systematic review of seven Italian reports. Ital J Pediatr. 2019;45(1):139. https://doi.org/10.1186/s13052-019-0736-5.

[14] Carbonell-Estrany X, Simões EAF, Dagan R, et al. Motavizumab for prophylaxis of respiratory syncytial virus in high-risk children: a noninferiority trial. *Pediatrics*. 2010;125(1):e35–51.

[15] Wang D, Cummins C, Bayliss S, Sandercock J, Burls A. Immunoprophylaxis against respiratory syncytial virus (RSV) with palivizumab in children: a systematic review and economic evaluation. Health Technol Assess. 2008;12(36):iii,ix-x,1–86. https://doi.org/10.3310/hta12360.

[16] Wang D, Bayliss S, Meads C. Palivizumab for immunoprophylaxis of respiratory syncytial virus (RSV) bronchiolitis in high-risk infants and young children: systematic review and additional economic modelling of subgroup analyses. Health Technol Assess. 2011;15(5):iii–iv,1–124. https://doi.org/10.3310/hta15050.

[17] Priante E, Tavella E, Girardi E, Militello MA, Mardegan V, Maule MM, et al. Restricted Palivizumab Recommendations and the Impact on RSV Hospitalizations among Infants Born at > 29 Weeks of Gestational Age: An Italian Multicenter Study. Am J Perinatol. 2019;36(S 02):S77–S82. https://doi.org/doi:10.1055/s-0039-1691771.

[18] Figueras-Aloy J, Carbonell-Estrany X, Quero-Jimenez J, Fernández-Colomer B, Guzmán-Cabañas J, Echaniz-Urcelay I, IRIS Study Group. FLIP-2 Study: risk factors linked to respiratory syncytial virus infection requiring hospitalization in

premature infants born in Spain at a gestational age of 32 to 35 weeks. Pediatr Infect Dis J. 2008;27:788–93. https://doi.org/10.1097/INF.0b013e3181710990.

19 Lanari M, Anderson EJ, Sheridan-Pereira M, et al. Burden of respiratory syncytial virus hospitalisation among infants born at 32–35 weeks' gestational age in the Northern Hemisphere: pooled analysis of seven studies. Epidemiol Infect. 2020;148:e170. https://doi.org/10.1017/S0950268820001661.

20 Notario G, Vo P, Gooch K, Deaton R, Wu X, Harris B, et al. Respiratory syncytial virus-related hospitalization in premature infants without bronchopulmonary dysplasia: subgroup efficacy analysis of the IMpact-RSV trial by gestational age group. Pediatric Health Med Ther. 2014;5:43–8. https://doi.org/10.2147/PHMT.S59572.

21 Chiou CF, Weaver MR, Bell MA, Lee TA, Krieger JW. Development of the multiattribute pediatric asthma health outcome measure (PAHOM). Int J Qual Health Care. 2005;17(1):23–30. https://doi.org/10.1093/intqhc/mzh086.
