# Supplementary material for: A new cost-utility analysis assessing risk factor-guided prophylaxis with palivizumab for the prevention of severe respiratory syncytial virus infection in Italian infants born at 29–35 weeks’ gestational age
Source: PLoS One. 2023 Aug 10;18(8):e0289828. doi: 10.1371/journal.pone.0289828 (PMC10414677; doi:10.1371/journal.pone.0289828)
Supplement: S5 Table — (PDF) [file pone.0289828.s006.pdf]

Table S5 Cost per QALY gained in moderate- and high-risk infants

	ICUR in indicated gestational age group		
**32-35wGA infants included in analysis**	**29-35wGA**	**29-31wGA**	**32-35wGA**
**High-risk**	€12311	€15139	€10825
**Moderate- and high- risk (base case)**	€14814	€15139	€14719
**Moderate-risk only**	€17799	€15139	€19541
