# Supplementary material for: A new cost-utility analysis assessing risk factor-guided prophylaxis with palivizumab for the prevention of severe respiratory syncytial virus infection in Italian infants born at 29–35 weeks’ gestational age
Source: PLoS One. 2023 Aug 10;18(8):e0289828. doi: 10.1371/journal.pone.0289828 (PMC10414677; doi:10.1371/journal.pone.0289828)
Supplement: S1 Fig — (PDF) [file pone.0289828.s007.pdf]

Figure S1 International Risk Scoring Tool – risk factors and scoring[1]

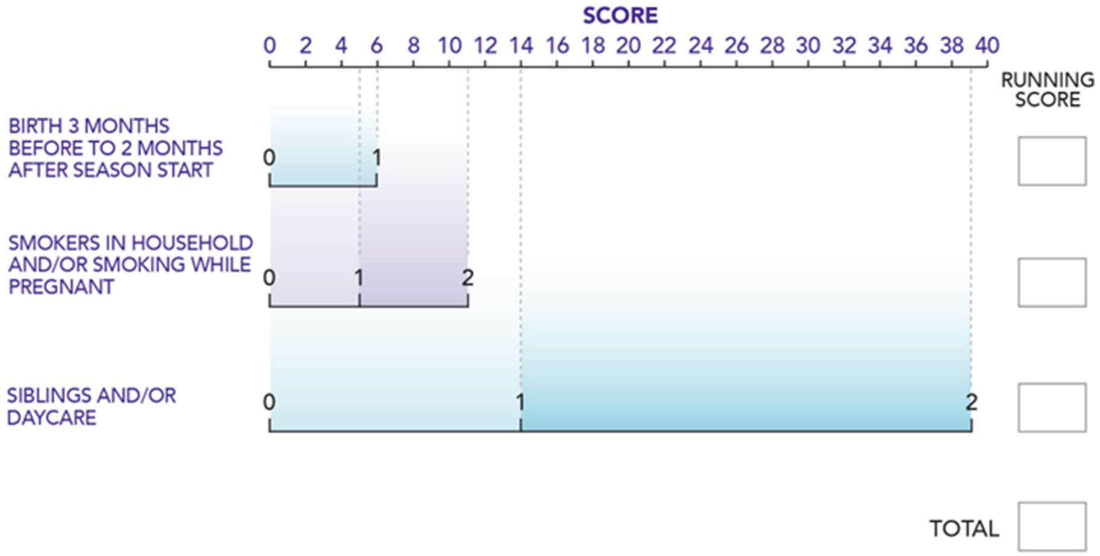

References

1 Blanken MO, Paes B, Anderson EJ, Lanari M, Sheridan-Pereira M, Buchan S, et al. Risk scoring tool to predict respiratory syncytial virus hospitalisation in premature infants. Pediatr Pulmonol. 2018;53(5):605–12. https://doi.org/10.1002/ppul.23960.
