# Supplementary material for: A new cost-utility analysis assessing risk factor-guided prophylaxis with palivizumab for the prevention of severe respiratory syncytial virus infection in Italian infants born at 29–35 weeks’ gestational age
Source: PLoS One. 2023 Aug 10;18(8):e0289828. doi: 10.1371/journal.pone.0289828 (PMC10414677; doi:10.1371/journal.pone.0289828)
Supplement: S2 Fig — (PDF) [file pone.0289828.s008.pdf]

Figure S2 One-way deterministic sensitivity analysis (±20%) for palivizumab *versus* no prophylaxis in the base case, 15 most sensitive variables

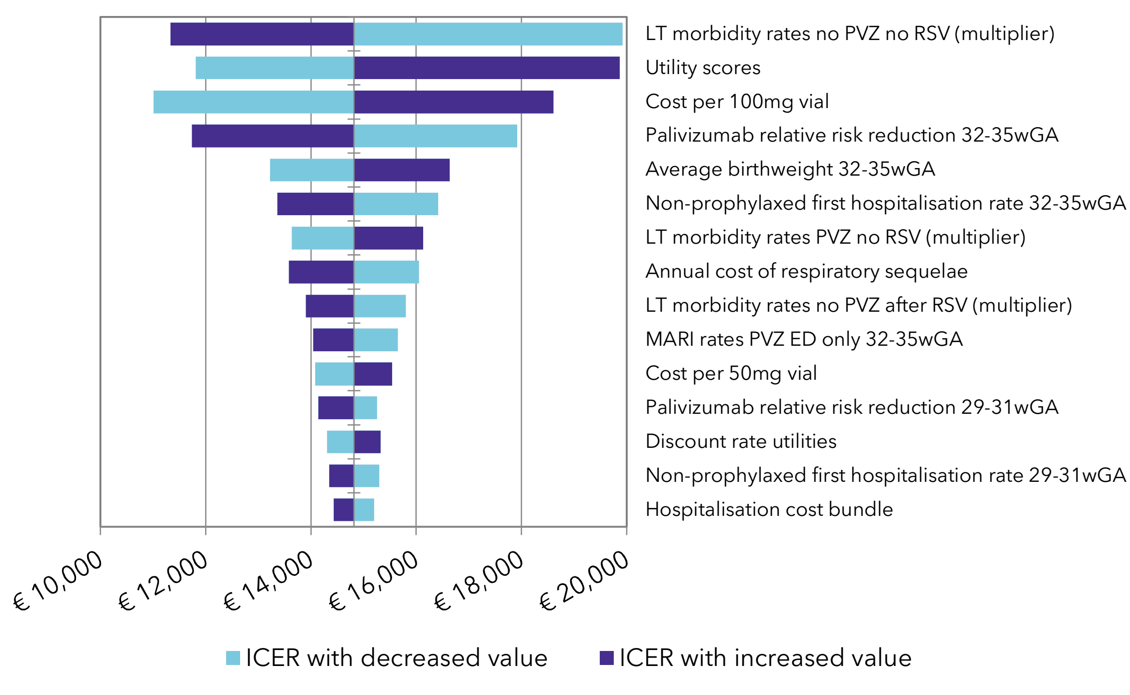

ED: emergency department; LT: long-term; mg: milligram; PVZ: palivizumab; RSV: respiratory syncytial virus; wGA: weeks' gestational age
